# DNA Methylation in the Neuropeptide S Receptor 1 (*NPSR1*) Promoter in Relation to Asthma and Environmental Factors

**DOI:** 10.1371/journal.pone.0053877

**Published:** 2013-01-23

**Authors:** Lovisa E. Reinius, Anna Gref, Annika Sääf, Nathalie Acevedo, Maaike Joerink, Maciej Kupczyk, Mauro D'Amato, Anna Bergström, Erik Melén, Annika Scheynius, Sven-Erik Dahlén, The BIOAIR Study Group, Göran Pershagen, Cilla Söderhäll, Juha Kere

**Affiliations:** 1 Department of Biosciences and Nutrition, Karolinska Institutet, Stockholm, Sweden; 2 Institute of Environmental Medicine, Karolinska Institutet, Stockholm, Sweden; 3 Department of Medicine Solna, Translational Immunology Unit, Karolinska Institutet, Stockholm, Sweden; 4 Astrid Lindgren Children's Hospital, Karolinska University Hospital, Stockholm, Sweden; 5 Science for Life Laboratory, Karolinska Institutet, Stockholm, Sweden; 6 Research Programs Unit, Biomedicum, University of Helsinki and Folkhälsan Institute of Genetics, Helsinki, Finland; 7 Centre for Allergy Research, Karolinska Institutet, Stockholm, Sweden; Ludwig-Maximilians-University Munich, Germany

## Abstract

Asthma and allergy are complex disorders influenced by both inheritance and environment, a relationship that might be further clarified by epigenetics. Neuropeptide S Receptor 1 (*NPSR1*) has been associated with asthma and allergy and a study suggested modulation of the genetic risk by environmental factors. We aimed to study DNA methylation in the promoter region of *NPSR1* in relation to asthma and environmental exposures. Electrophoretic Mobility Shift Assay (EMSA) was used to investigate potential functional roles of both genotypes and methylation status in the *NPSR1* promoter. DNA methylation was analysed using EpiTYPER in blood samples from two well-characterized cohorts; the BIOAIR study of severe asthma in adults and the Swedish birth cohort BAMSE. We observed that DNA methylation and genetic variants in the promoter influenced the binding of nuclear proteins to DNA, suggesting functional relevance. Significant, although small, differences in methylation were related to both adult severe asthma (*p* = 0.0001) and childhood allergic asthma (*p* = 0.01). Furthermore, DNA methylation was associated with exposures such as current smoking in adults for two CpG sites (*p* = 0.005 and 0.04), parental smoking during infancy in the children (*p* = 0.02) and in which month the sample was taken (*p* = 0.01). In summary, DNA methylation levels in the promoter of *NPSR1* showed small but significant associations with asthma, both in adults and in children, and to related traits such as allergy and certain environmental exposures. Both genetic variation and the methylated state of CpG sites seem to have an effect on the binding of nuclear proteins in the regulatory region of *NPSR1* suggesting complex regulation of this gene in asthma and allergy.

## Introduction

Asthma and allergy are complex disorders influenced by both inheritance and environment. In recent decades there has been a marked increase in their prevalence that today has reached a plateau, a development that cannot be explained by genetics alone. Epigenetics might help to explain this development of asthma and allergy, but studies on this topic are so far few. DNA methylation is an epigenetic modification that occurs mostly on cytosines followed by guanines, so called CpG sites. There are approximately 28 million such CpG sites in the human genome and of these, 70 to 80% are methylated [1]. Methylation of DNA has previously been shown to regulate gene expression [2] and it is believed that the occurrence of these marks/modifications can be affected by the environment and that this could partly explain the increase in the prevalence of asthma and allergy [3]. For example, exposure to smoking *in utero* may influence DNA methylation in the offspring [4], and has been associated with an increased risk of childhood asthma [5], [6]. This effect was also observed in grandchildren of grandmothers that smoked during the mother's fetal period which indicates a transgenerational effect that might be mediated by a change in DNA methylation patterns.

The Neuropeptide S Receptor 1 (*NPSR1*) gene on chromosome 7p14.3 encodes a G-protein coupled receptor that has been associated to many complex disorders such as asthma, irritable bowel syndrome, rheumatoid arthritis and psychiatric disorders [7]–[12]. The receptor was first identified as an asthma candidate gene by positional cloning and has since then been replicated several times in different ethnic populations [10], [13]–[20]. Recent data suggest that the genetic background of *NPSR1* can modify the effect of environmental factors such as farm exposure on the development of allergic symptoms [21]. The underlying mechanism of this effect in asthma is still unknown.

Since *NPSR1* is a strong candidate gene for asthma and one study suggested that its genetic risk is modulated by the environment, we aimed to study DNA methylation in the regulatory region of *NPSR1* in relation to asthma and environmental exposures. We used Electrophoretic Mobility Shift Assay (EMSA) to investigate a potential functional role of both genotypes and methylation status in the regulatory region of *NPSR1*. We used two well characterized cohorts that offer unique opportunities to investigate genetics and epigenetics in relation to asthma phenotypes and environmental exposures; the BIOAIR study of severe asthma in adults [22] and the Swedish birth cohort BAMSE [23].

## Results

### Transcription factor binding sites, DNA methylation, and genetic variants

**Figure 1:**
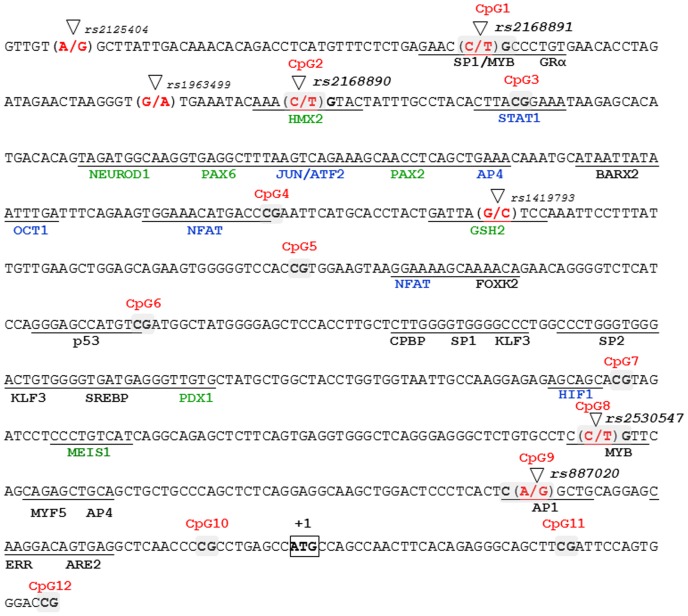
The promoter region of the Neuropeptide S Receptor 1 gene (*NPSR1*). Polymorphisms are marked by triangles, transcription factor binding sites are underlined and CpG sites are shaded in gray. Green color of the transcription factor indicates neurologically associated factors, blue indicates immunologically associated factors, and black indicates general factors.

**Figure 2:**
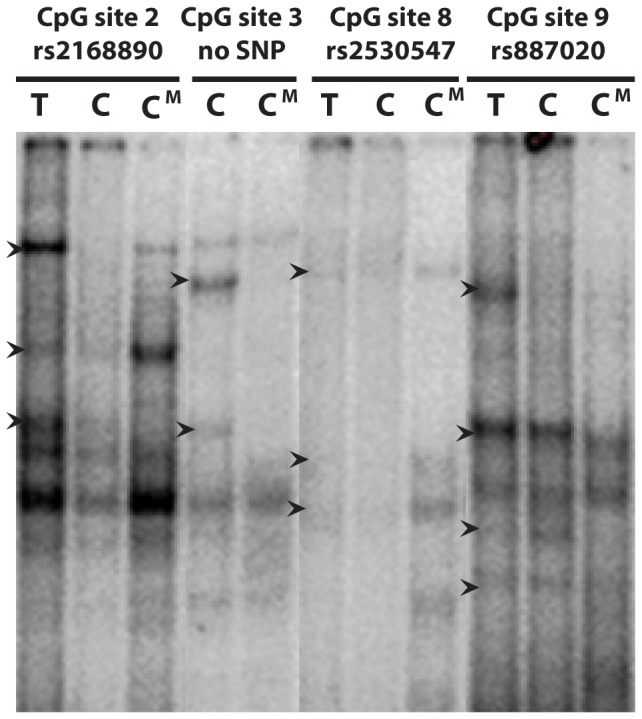
Electric Mobility Shift Assay (EMSA) for four sites in the promoter for Neuropeptide S Receptor 1 gene (*NPSR1*). The experiment was performed three separate times using nuclear protein extracts from two different cell types (Colo205 and HEK293). Data presented here is a representative gel using nuclear extract from Colo205. Data was similar for nuclear cell extracts from both cell lines. The sites studied included CpG site 2 coinciding with rs2168890 in a predicted HMX2 binding site, CpG site 3 in a predicted STAT1 binding site, CpG site 8 coinciding with rs2530547 in a predicted binding site for MYB, and CpG site 9 coinciding with rs887020 in a predicted binding site for AP1. Arrows indicates sites showing differential binding.

The *NPSR1* promoter contains twelve CpG sites (Figure 1) [24]. To evaluate potential functionality of these CpG sites, we performed EMSA to test for possible differential protein binding based on both genotype and methylation status. Four suggestive transcription factor binding sites in the promoter were selected based on the presence of a CpG site within the predicted binding site. The sites studied included CpG site 2 coinciding with rs2168890 in a predicted Hmx2 binding site, CpG site 3 in a predicted STAT1 binding site, CpG site 8 coinciding with rs2530547 in a predicted binding site for Myb, and CpG site 9 coinciding with rs887020 in a predicted binding site for AP1 (Figure 1). EMSA using nuclear extracts from both Colo205 and HEK293 cells showed clear differences in binding of nuclear proteins based both on genotype and methylation status of the C allele in the CpG site 2/rs2168890 (Figure 2). CpG site 3 showed differences in binding of nuclear proteins between unmethylated and methylated cassettes with loss of bands for the methylated cassette. The results for CpG site 8 showed no or very weak binding for either allele of the SNP rs2530547 while the methylated C allele bound nuclear proteins. CpG site 9 showed loss of protein binding for the C allele, both methylated and unmethylated, as compared to the T allele (*upper band*, Figure 2). Taken together, the EMSA results suggest that DNA methylation, in the promoter of *NPSR1*, could functionally be relevant based on the differential binding of transcription factors due to not only genetic variation but also the methylated state of the allele creating a CpG site.

### Variation of DNA methylation in main blood cell types

**Figure 3:**
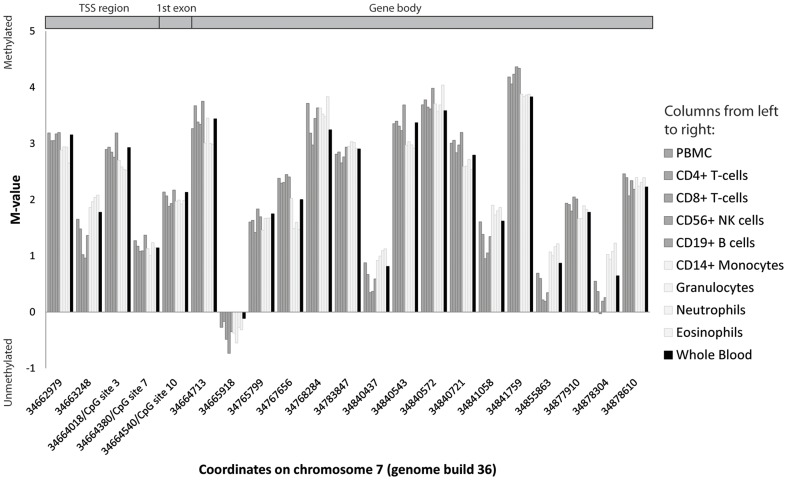
Neuropeptide S Receptor 1 gene (*NPSR1*) DNA methylation levels in isolated cells from peripheral blood. Peripheral blood donations were obtained from six healthy adult male donors. Data were obtained using the Illumina Infinium 450K bead array and the M-value represents methylation degree [25]. Dark grey bars represent lymphoid cells, light grey bars myeloid cells and black bar whole blood. TSS – Transcription start site.

To assess whether it would be meaningful to measure DNA methylation levels in peripheral blood samples, we compared the degree of methylation in the *NPSR1* region in a set of samples of whole blood and fractionated white blood cells from six healthy adult male donors [25]. Three of the probes included in the Illumina Infinium 450K array for DNA methylation were overlapping with CpG sites in the *NPSR1* promoter region analyzed here, *i.e.* CpG sites 3, 7 and 10, described below. The results show that across *NPSR1*, the degree of methylation in different types of white blood cells is relatively constant, suggesting that methylation measurements are reliable for *NPSR1* in whole blood irrespective the differential count of white blood cells (Figure 3). Furthermore, to get a more complete picture of the cell specific DNA methylation pattern in the *NPSR1* promoter region, we use the EpiTYPER technology to determine DNA methylation levels of CpG site 1–12 in the same samples used for the array based method (Figure S5). Again, we confirm that the degree of methylation is similar across different cell types in whole blood for the promoter region studied in *NPSR1*.

### DNA methylation and genetic variation in the NPSR1 promoter

We next performed a pilot study to evaluate if differences in DNA methylation status can be identified in whole blood from patients with asthma and other obstructive airway disease, including adults with mild asthma, severe asthma or chronic obstructive pulmonary disease (COPD) from the BIOAIR study (n = 171, Table 1) and a subset of 260 randomly selected children with or without asthma from the BAMSE birth cohort. DNA methylation levels for twelve CpG sites in the promoter region of *NPSR1*, approximately 600 base pairs 5′ of the transcription start site (Figure 1) were analysed using EpiTYPER [26]. There was a difference in both age and gender distribution for the adults in the BIOAIR study. Patients with COPD were older and consisted of more males compared to both mild and severe asthmatics (Table 1). The age and gender distributions among healthy and asthmatic children were similar between those 260 randomly chosen from the BAMSE birth cohort (healthy children 8.3±0.4 years and 52% boys, asthmatic children 8.4±0.4 years and 61% boys). The difference between minimum and maximum measured levels in DNA methylation for all the CpG sites in the promoter region (Δ_max-min_) varied from lowest 15% to highest 47% in the total adult sample set and the total childhood sample set varied from lowest 10% to highest 52%. Most CpG sites in the promoter showed very high levels of average methylation (>75%) with the exception of CpG site 8 showing lower methylation levels (<60%). There were indications of difference in DNA methylation based on adult asthma and other obstructive airway disease for CpG site 2 (homozygous C allele carriers only) and CpG site 5 in the promoter region (Table S1). None of the CpG sites analysed for the smaller selection of BAMSE samples (n = 260) were differentially methylated between the groups but they showed overall similar degree of methylation as compared to the adult BIOAIR samples (Table S1).

**Table 1:** Characteristics of the BIOAIR study population (n = 171).

	Mild Adult Asthma	Severe Adult Asthma	COPD	p-value
n	56	67	48	
Age (years)	41.6±13.2	50.3±13.2	63.8±8.5	<0.05
Gender (% males)	44.6	43.3	75	<0.05
BMI (kg/m^2^)	25.2±3.8^1^	28.7±5.3	27.4±5.3^1^	<0.05
Current smokers (n = 14)	1	2	11	<0.05
Former smokers (n = 67)	12	18	37	
FEV_1_ (%pred)	87.3±18.3	71.4±21.4	50.0±16.1	<0.05
FVC (%pred)	104.6±16.1	87.5±20.6^1^	77.2±16.5	<0.05
Blood eosinophils (10^9^/L)	0.31±0.27^2^	0.37±0.35^3^	0.23±0.29^3^	ns
rs2168891 CC/CT/TT (%)	83.9/14.3/1.8	83.6/16.4/0	91.7/8.3/0	ns
rs2168890 CC/CT/TT (%)	69.1/30.9/0^1^	71.4/28.6/0^4^	73.3/26.7/0^5^	ns
rs2530547 CC/CT/TT (%)	29.1/54.5/16.4^1^	40.9/45.5/13.6^1^	40.4/48.9/.10.6^1^	ns
rs887020 AA/AG/GG (%)	28.6/53.6/17.9	17.9/52.2/29.9	20.8/58.3/20.8	ns

COPD - Chronic Obstructive Pulmonary Disease, BMI – Body Mass Index, FEV_1_ – Forced Expiratory Volume in 1 second, FVC – Forced Vital Capacity. Data are presented as mean ± standard deviation. P-values were calculated using ANOVA for continuous variables and chi square test for distributions.

1 one individual with missing data,

2 five individuals with missing data,

3 two individuals with missing data,

4 seven individuals with missing data,

5 three individuals with missing data.

Next, we investigated whether SNPs located in the predicted *NPSR1* promoter region were associated with asthma and other obstructive airway disease in the 171 adult samples from BIOAIR and the 260 BAMSE children. Four SNPs were genotyped, rs2168891, rs2168890, rs2530547 and rs887020, which coincide with CpG sites 1, 2, 8 and 9, respectively (Figure 1). The genotype distribution for the four SNPs were similar between the groups (mild asthma *vs.* severe asthma *vs.* COPD or healthy *vs.* asthmatic children) showing no significant association with asthma in neither the BIOAIR study nor the BAMSE randomised children (data not shown). Importantly, we found that the level of DNA methylation was affected by genotypes in the predicted *NPSR1* promoter region. For example, at CpG site 2 (rs2168890) and CpG site 8 (rs2530547), a step-wise decrease in DNA methylation was observed depending the CC, CT or TT genotype, respectively. However, there were no effects on DNA methylation neither for the rs2168891 at CpG site 1 nor for the rs887020 at CpG site 9. Only homozygous carriers of the allele creating a CpG site were considered for further analysis both in the BIOAIR and the BAMSE analysis.

### NPSR1 promoter methylation status, asthma and other obstructive airway disease, lifestyle and exposure in adults

The BIOAIR study is an extensive collection of data including a one year follow up of the patients [22] and was therefore sought to be investigated in more detail with the respect to DNA methylation, asthma and environmental exposures. The effect of phenotypes related to asthma and COPD and lifestyle factors (including BMI and smoking) on DNA methylation of the predicted *NPSR1* promoter region (CpG site 1–12), are shown in Table 2. For analyses regarding the CpG site 1, 2, 8 and 9, only homozygous individuals for the allele creating the CpG site were included due to confounding effects of the SNP on the methylation analysis (n = 147, 116, 62 and 40, respectively). An adjusted linear regression model was used to identify associations between DNA methylation and asthma and other obstructive airway disease. There was significantly decreased methylation levels for CpG site 5 in severe asthma patients compared to mild asthma patients when adjusting for age, gender and country of origin (*p* = 0.0001, Table 2). Current smoking showed largest effects on DNA methylation levels. CpG site 1 showed significantly increased levels of methylation in relation to current smoking (*p* = 0.005) but not to former smoking (*p* = 0.13). For CpG site 8, the effect of current smoking resulted in 8% change in DNA methylation (*p* = 0.042) while former smoking resulted in a 6% change (*p* = 0.013, Table 2). Small but significant changes in the level of DNA methylation were also linked to BMI for CpG site 2, 3 and 5 and to age for CpG site 4 (*p* = 0.045, Table 2). Given the known relationship between asthma and obesity/BMI, we also adjusted the analyses between methylation status at CpG site 5 and severe asthma for BMI and found the results unchanged (p = 0.002). However, the association between CpG site 5 and BMI became non-significant (p = 0.12) after adjustment for asthma status, which indicates that this effect at CpG site 5 is mediated by asthma status rather than BMI per se. Results were unchanged between BMI and CpG sites 2 and 3 after adjustment for affection status. There were no relationships found between lung function tests (FEV_1_ and FVC) or blood eosinophil counts and DNA methylation.

**Table 2:** The effect of respiratory phenotypes and lifestyle on the levels of DNA methylation in the *NPSR1* promoter region covering CpG site 1 to 12 in adults from the BIOAIR study (n = 171) using adjusted linear regression models.

	CpG site 1*	CpG site 2**	CpG site 3	CpG site 4	CpG site 5	CpG site 6	CpG site 8†	CpG site 9††	CpG site 10	CpG site 11_12
**Methylation level min-max (%)**	75.7–100	62.1–95.2	71.9–100	64.4–92.2	71.0–100	60.2–100	24.7–71.3	77.6–92.3	56.1–100	70.9–92.2

	Methylation level, difference in %	P	Methylation level, difference in %	P	Methylation level, difference in %	P	Methylation level, difference in %	P	Methylation level, difference in %	P	Methylation level, difference in %	P	Methylation level, difference in %	P	Methylation level, difference in %	P	Methylation level, difference in %	P	Methylation level, difference in %	P
**Severe asthma**	1.05	0.25	2.67	0.40	−1.20	0.25	−0.76	0.44	−3.29	0.0001	−1.11	0.54	0.15	0.96	0.20	0.89	2.18	0.14	0.28	0.73
**COPD**	1.27	0.26	3.12	0.43	−0.51	0.71	−0.31	0.81	−1.95	0.089	−1.33	0.56	6.03	0.12	1.05	0.63	1.14	0.54	1.26	0.24
**Age (years)**	−0.05	0.12	−0.11	0.35	−0.050	0.17	−0.069	0.045	0.041	0.19	0.037	0.54	−0.15	0.16	0.034	0.52	−0.0028	0.95	0.010	0.72
**Gender (male/female)**	−0.14	0.86	1.85	0.51	0.52	0.58	−1.00	0.25	0.24	0.77	−0.70	0.65	1.58	0.53	0.71	0.57	0.79	0.53	0.80	0.27
**BMI (kg/m^2^)**	−0.10	0.19	−0.62	0.027	−0.23	0.007	0.075	0.36	−0.18	0.019	−0.040	0.78	−0.27	0.40	−0.067	0.68	−0.037	0.76	−0.11	0.095
**Current smoking**	3.80	0.005	3.08	0.57	1.56	0.35	0.27	0.86	−0.55	0.71	1.18	0.66	8.74	0.042	1.54	0.55	−0.62	0.78	−0.69	0.59
**Former smoking**	−1.23	0.13	4.04	0.16	−0.76	0.43	1.36	0.14	−0.14	0.87	−0.23	0.89	6.36	0.013	−1.59	0.22	−1.79	0.19	0.14	0.86
**FEV_1_ (% pred)**	−0.030	0.083	0.11	0.086	0.0080	0.69	−0.0018	0.93	0.017	0.33	−0.011	0.76	0.012	0.86	−0.048	0.11	0.011	0.70	−0.0088	0.59
**FVC (% pred)**	−0.021	0.28	0.099	0.18	0.00018	0.99	−0.017	0.41	0.023	0.25	–0.030	0.43	0.064	0.41	–0.045	0.19	–0.0044	0.89	–0.0091	0.60
**Blood Eosinophil (10^9^/L)**	0.31258	0.80	–1.10	0.73	–0.095	0.95	0.40	0.76	0.22	0.86	2.33	0.32	–2.04	0.57	1.11	0.61	1.98	0.32	–1.18	0.28

COPD – Chronic Obstructive Pulmonary Disease, BMI – Body Mass Index, FEV_1_ – Forced Expiratory Volume in 1 second, FVC – Forced Vital Capacity. Mild asthma patients were used as reference in the comparison to severe asthma and COPD. P: p-value for linear regression model adjusted for age, gender and country of origin. Age and gender were also adjusted for asthma and other obstructive airway disease (mild asthma, severe asthma or COPD).

* Data analyzed in homozygous carriers of the C allele only (n_mild asthma/severe asthma/COPD_ = 47/56/44),

** Data analyzed in homozygous carriers of the C allele only (n_mild asthma/severe asthma/COPD_ = 38/45/33),

† Data analyzed in homozygous carriers of the C allele only (n_mild asthma/severe asthma/COPD_ = 16/27/19),

†† Data analyzed in homozygous carriers of the G allele only (n_mild asthma/severe asthma/COPD_ = 10/20/10).

### NPSR1 promoter methylation status and asthma in children

Based on the results from EMSA as well as the suggestive associations between asthma and life style factors, and DNA methylation in the 5′ end of the predicted *NPSR1* promoter for the BIOAIR study, we analysed CpG sites 1 to 5 more in depth in a larger number of children from the BAMSE study (n = 546). The children were of the same age but gender distribution differed among healthy and asthmatic children and asthmatic children were more frequently exposed to parental smoking in infancy (Table 3). To answer the question if methylation levels were associated with childhood asthma, adjusted linear regression was performed between methylation status and different asthma outcomes. For analyses regarding the CpG site 1 and 2, only homozygous individuals for the allele creating the CpG site were included due to confounding effects of the SNP on the methylation analysis (n = 470 and 397, respectively). Four models were considered to test for differences in methylation status between healthy controls and cases diagnosed with: 1) asthma ever up to 8 years, 2) allergic asthma up to 8 years, 3) non-allergic asthma up to 8 years, and 4) current asthma at 8 years. The results showed a small, but significantly lower *NPSR1* promoter methylation level in children diagnosed with asthma ever up to age 8 as compared to healthy children at CpG site 4 (*p* = 0.04, Table 4). Lower DNA methylation levels of the promoter were also seen in children with allergic asthma at CpG site 1 (*p* = 0.01) and at CpG site 4 (*p* = 0.04) as compared to healthy children, whereas no differences were seen between non-allergic asthmatics and healthy children. The level of significance remained after adjustment for gender, age at blood sampling and plate used for sodium bisulfite treatment. The results showed no significant difference in *NPSR1* promoter methylation levels between children with current asthma as compared to healthy children. CpG site 5 was not associated with any asthma outcome in children.

**Table 3:** Characteristics of the BAMSE study population (n = 546).

	Controls	Asthma ever	p-value
n	273	273	
Age (years)	8.3±0.5	8.4±0.5	ns
Gender (% boys)	48.4	61.5	<0.05
rs2168891 CC/CT/TT (%)	89.0/11.0/0^1^	88.4/11.6/0^2^	ns
rs2168890 CC/CT/TT (%)	77.3/21.9/0.8^3^	76.9/22.4/0.8^4^	ns
rs2530547 CC/CT/TT (%)	46.9/40.8/12.2^5^	38.6/47.9/13.5^2^	ns
rs887020 AA/AG/GG (%)	16.8/48.4/34.8^6^	21.4/50.8/27.8^7^	ns
Allergic asthma	0	136	na
Non-allergic asthma	0	137	na
Current asthma	0	123	na
Parental smoking in infancy (%)	17.7	26.1	<0.05
Current parental smoking (%)	17.0	22.6	ns
Current dietary folate intake ( µg/day)	217.0±36.9	214.2±31.4	ns
Current BMI (kg/m^2^)	17.1±1.8	17.3±2.0	ns
Sampling season of blood samples			ns
January-March	87	86	
April-June	78	87	
July-September	40	38	
October-December	68	62	

BMI – Body Mass Index, ns – non significant, na – not analyzed. Data are presented as mean ± standard deviation. P-values were calculated using Student's T-test for continuous variables and chi square test for distributions.

1 ten individuals with missing data,

2 six individuals with missing data,

3 13 individuals with missing data,

4 18 individuals with missing data,

5 11 individuals with missing data,

6 29 individuals with missing data,

7 25 individuals with missing data.

**Table 4:** DNA methylation status compared between asthma cases and controls in the BAMSE cohort (n = 546) using adjusted linear regression models.

	Case (n)	CpG site 1*			CpG site 2**			CpG site 3			CpG site 4			CpG site 5		
	Control (n)	% mean	P†	P††	% mean	P†	P††	% mean	P†	P††	% mean	P†	P††	% mean	P†	P††
**Methylation level (min-max %)**		83.7–98.2			61.8–92.4			66.6–98.3			66.5–88.5			67.8–96.6		
**Asthma ever**	case (273)	91.1	0.07	0.08	80.7	0.81	0.93	85.9	0.55	0.53	80.9	0.04	0.05	86.3	0.84	0.79
	control (273)	91.4			80.8			86.2			81.4			86.2		
**Allergic asthma**	case (136)	90.8	0.01	0.01	80.2	0.27	0.39	85.6	0.31	0.13	80.8	0.04	0.03	86.1	0.67	0.72
	control (273)	91.4			80.8			86.1			81.4			86.2		
**Non-allergic asthma**	case (137)	91.3	0.79	0.62	81.1	0.51	0.35	86.2	0.96	0.81	81.0	0.21	0.20	86.5	0.45	0.44
	control (273)	91.4			80.8			86.2			81.4			86.2		
**Current asthma**	case (123)	91.2	0.31	0.30	80.7	0.91	0.79	85.8	0.49	0.48	80.9	0.09	0.11	86.8	0.13	0.14
	control (273)	91.4			80.8			86.2			81.4			86.2		

* Only homozygous carriers of the allele (rs2168891) creating a CpG site were included in the analyses of CpG site 1 (Asthma ever n _cases/controls_ = 236/234, Allergic asthma n _cases/controls_ = 120/234, Non-allergic asthma n _cases/controls_ = 116/234, Current asthma n _cases/controls_ = 103/234).

** Only homozygous carriers of the allele (rs2168890) creating a CpG site were included in the analyses of CpG site 2 (Asthma ever n _cases/controls_ = 196/201, Allergic asthma n _cases/controls_ = 94/201, Non-allergic asthma n _cases/controls_ = 201/201, Current asthma n _cases/controls_ = 89/201).

† p-value for crude linear regression model.

†† p-value for linear regression model adjusted for gender, age at blood sampling and plate used for sodium-bisulfite treatment.

### NPSR1 promoter methylation status, lifestyle and environmental factors in children

Extended analyses of the 5′end of the predicted *NPSR1* promoter was also performed to test if the methylation status was affected by lifestyle and environmental factors in children up to 8 years of age (Table 5). Parental smoking in infancy was associated with a small, but significantly lower level of DNA methylation in CpG site 1 (*p* = 0.02, Table 5). However, there was no significant association to current parental smoking. High current dietary folate intake was associated with decreases in DNA methylation levels at CpG site 1 (*p* = 0.01) and CpG site 4 (*p* = 0.04). No association was observed in *NPSR1* promoter methylation in relation to exposure to traffic-NO_x_ during infancy or currently, or to current BMI (Table 5). Interestingly, a seasonal effect was found on the DNA methylation status of the predicted *NPSR1* promoter region. A significant increase in DNA methylation of CpG site 5 was observed for samples obtained during July to September (*p* = 0.01), and October to December (*p* = 0.02), as compared to January to March, which was used as reference category. After additional adjustment for birth month, the significance remained at CpG site 5 July to September (*p* = 0.003). Further analyses showed that sensitization to any inhalant allergen (positive Phadiatop) was associated with decreased methylation in CpG site 1 (p = 0.01), similar to the results for allergic asthma (Table 4). Specifically, sensitization to birch and dog were associated with methylation changes (p = 0.05 and 0.04, respectively).

**Table 5:** The effect of lifestyle and environmental factors on the levels of DNA methylation in the *NPSR1* promoter region in children from the BAMSE study (n = 546) using adjusted linear regression models.

	CpG site 1*	CpG site 2**	CpG site 3	CpG site 4	CpG site 5
**Methylation level (min-max %)**	83.7–98.2	61.8–92.4	66.6–98.3	66.5–88.5	67.8–96.6

	Methylation level, difference in %	P	Methylation level, difference in %	P	Methylation level, difference in %	P	Methylation level, difference in %	P	Methylation level, difference in %	P
**Parental smoking in infancy**	−0.52	0.02	−0.28	0.55	0.04	0.94	−0.18	0.53	−0.08	0.83
**NO_x_ from traffic in infancy** †	−0.13	0.80	−1.06	0.31	−0.44	0.72	−0.22	0.74	−0.45	0.58
**Current parental smoking**	0.18	0.44	0.04	0.93	−0.61	0.26	−0.09	0.76	−0.60	0.12
**Current NO_x_ from traffic** †	−0.15	0.64	0.05	0.94	0.18	0.80	−0.44	0.26	−0.24	0.63
**Current dietary folate intake** ††	−0.70	0.01	−0.02	0.97	−0.32	0.61	−0.71	0.04	−0.53	0.22
**Current BMI (kg/m^2^)**	0.01	0.90	−0.15	0.13	−0.04	0.69	0.00	0.96	−0.06	0.43
**Sampling season**										
**January-March (ref)**	90±1.9		83±3.9		94±4.5		82±2.5		90±3.1	
**April-June**	−0.26	0.37	−0.55	0.34	−0.67	0.31	−0.51	0.16	0.11	0.80
**July-September**	−0.39	0.26	−1.37	0.06	0.62	0.44	0.00	1.00	1.38	0.01
**October-December**	−0.31	0.30	−0.10	0.86	0.16	0.81	0.12	0.73	1.11	0.02

* Children not homozygous for major allele of SNP in CpG site 1 (rs2168891) were excluded from CpG site 1 analyses.

** Children not homozygous for major allele of SNP in CpG site 2 (rs2168890) were excluded from CpG site 2 analyses. P: p-value for linear regression model adjusted for gender, age at blood sampling and plate used for sodium-bisulfite treatment.

† Data analyzed based on the 5^th^–95^th^ percentile difference in NO_x_ ( µg/m^3^) exposure levels, additional adjustment made for municipality.

†† Data analyzed based on highest versus lowest quartile (Q1 versus Q4).

## Discussion

This study presents an extensive analysis of DNA methylation in the promoter of *NPSR1* in relation to asthma, lifestyle and exposures in both adults and children. We found a functional relevance of DNA methylation at certain CpG sites in the promoter based on differential binding of nuclear proteins, an effect also shown for genetic variants. Furthermore, there were small but significant differences in DNA methylation in relation to adult severe asthma (but not COPD) as well as to allergic asthma in children. Furthermore, we observed associations between DNA methylation changes in the promoter region and current smoking in adults and passive smoking in children. The previous genetic associations seen to IgE levels and allergy in relation to asthma for *NPSR1* polymorphisms [10], [14], [20], [27], in combination with our results in this study, highlight the importance this gene might have in the corresponding biology.

Many of the genetic associations concerning *NPSR1* and asthma are related to allergic asthma [10], [14], [20], [27]. Up-regulation of *Npsr1* expression in the lung has been observed in one mouse model of allergen-induced airway inflammation [10] but not in other models [28], [29]. Low levels of methylation are generally associated with higher levels of gene expression [2]. We found that DNA methylation in the predicted *NPSR1* promoter was slightly lower in peripheral blood from children that have allergic asthma as compared to healthy children. Although it remains to compare methylation patterns in blood and lung tissue specimens from subjects with asthma, recent data suggest that the pattern in blood inflammatory cells reflect those in the lung tissue [30]. It should be noted that parental report of doctor's diagnosis of asthma was used as a basis for all asthma phenotypes in BAMSE, and that the cohort data did not allow for analyses of e.g. severe asthma in children as evaluated by a paediatrician. Further studies on other asthma phenotypes such as severe asthma are warranted.

We identified methylation sites in the promoter for *NPSR1* that showed differential methylation in relation to exposures of smoking, either current smoking in adults or passive smoking in children. Several studies have shown effects of smoking on DNA methylation, both on candidate gene level and globally [31]–[34]. Interestingly, a previous report from the BAMSE birth cohort found a dose-response relation between exposure to parental smoking in infancy and IgE sensitisation [35], supporting a link between variations in *NPSR1* methylation and allergic asthma.

To further support an effect between DNA methylation in *NPSR1* and allergic asthma, we saw an increase in DNA methylation related to blood sampling during the months July to December compared to January to March in both asthmatic and healthy children. Seasonal variation has been shown to influence DNA methylation at certain genomic regions when considering prenatal nutritional variation during periods of famine [36], [37]. Our data may indicate an environmental effect of, for example, allergens such as airborne pollen exposure during spring and/or a greater exposure to certain viruses and other infections during winter time, both relevant factors for the exacerbation of asthma. Further studies are however needed to clarify the role of seasonal effects on methylation patterns.

A methyl-rich diet can affect methylation status and enhance allergic airway disease in mice offspring [38], and periconceptional maternal folic acid use is associated with increased methylation in the growth promoting gene *IGF2* [39]. Here we report a decreased methylation level in the predicted *NPSR1* promoter in association with high dietary folate intake in children. The conflicting results may partly be explained by the fact that we consider a daily intake of folate from the diet whereas others have looked at intake from supplements. Further, compared to others we have investigated the child's current intake which might not affect methylation to the same degree as for example intrauterine exposure. Nevertheless, our results should be interpreted with caution until further replicated.

Traffic related air pollution exposure has been linked to incident airway disease in a previous study in the BAMSE birth cohort [40] and this effect appeard to be modified by genetic polymorphisms [41]. Traffic related air pollution has also been reported to affect DNA methylation [42], [43]. We found no effect between NO_x_ from traffic and methylation in the promoter of *NPSR1*. The NO_x_ levels in Stockholm are comparatively low in an international comparison, which may contribute to the absence of association.

In this study, we have focused on asthma and used DNA derived from peripheral blood as target for analysis. Importantly, there are several tissues expressing *NPSR1* mRNA and protein such as immune cells, gut, skin and lung epithelium as well as the brain [9], [10], [44]–[47]. Furthermore, *NPSR1* has not only been associated to asthma and related phenotypes but also to other diseases and target tissues. While the expression of *NPSR1* is rather low in immune cells, in accordance with our finding of high levels of DNA methylation, other tissues might show larger variations in both expression and DNA methylation and therefore further, more detailed studies in various tissues are required in the future. However, even though peripheral blood is a mixture of many cell types in different developmental stages we could here show that the cell specific patterns for DNA methylation regarding *NPSR1* in blood show little variation indicating that using whole blood could still be relevant. Also, although our data suggest that the region we have studied is a functional promoter [24], other alternative promoters may exist that could show cell specificity.

Age is a factor that long has been associated with changes in DNA methylation [48], [49]. It has been shown that genome-wide DNA methylation generally decreases with age [48], [50] but certain sites also show increased methylation [48]. Furthermore, the rate of methylation changes in blood may be higher in children compared to adult populations [48]. In our study we saw some effects by age on the levels of DNA methylation for certain CpG sites, specifically for CpG site 3. Here, younger study participants showed lower levels of methylation when comparing all adults to all children irrespective of disease (Figure S1).

In summary, DNA methylation levels in the promoter of *NPSR1* showed significant associations with asthma and related traits such as allergy, as well as with certain exposures, when measured in DNA from whole blood, although absolute differences were generally small. Importantly, EMSA performed in the same region suggest that both genetic variation and the methylated state of the CpG sites have an effect on the binding of transcription factors in the regulatory region of *NPSR1*. These results, however, need to be further evaluated in other target tissues and/or specific cell types in an independent sample material.

## Materials and Methods

### Ethical statement

This study was performed in accordance with the principles expressed in the Declaration of Helsinki. The study was approved by the local ethics committee at Karolinska Institutet and the BIOAIR study (clinicaltrials.gov.NCT00555607; EU Contract QLG1-CT-2000-01185) was approved by the ethics review boards and drug regulatory authorities in the twelve participating centers (Stockholm, Athens, Heraklion, Gent, Montpellier, London, Southampton, Krakow, Palermo, Ferrara, Hamburg and Leiden). Written informed consent for genetic testing was obtained either by the adult study participant or from the parents of the participating children.

### Study design

This study was conducted in four steps: 1) a functional study to evaluate the relevance of DNA methylation in the predicted *NPSR1* promoter, 2) testing of DNA methylation at CpG sites in the promoter region of *NPSR1* in a smaller set of human DNA samples (both adults and children) obtained from peripheral blood, and 3) general analysis of all CpG sites in the promoter in relation to respiratory phenotypes, life style factors and exposures in the BIOAIR study [22], 4) in depth analysis of CpG sites shown in previous steps to be of interest for further analysis in a larger data set from the BAMSE birth cohort [23] which would allow for studying the influence of environmental exposures.

#### BIOAIR

The aim of BIOAIR (the BIOmarkers of severe chronic AIRways disease) study is to clarify the natural course of severe asthma, elucidate mechanisms, risk factors, markers of severity, exacerbations and poor outcome [22]. Adult patients with mild controlled asthma, severe uncontrolled asthma and chronic obstructive pulmonary disease (COPD) were followed for one year. Data were collected at baseline and at several visits during one year of follow up and included: spirometry (FIV_1_, FEV_1_, FVC, FEV_1_/FVC), clinical characteristics, medical history, induced sputum, urine, medication use and questionnaires (respiratory quality of life and asthma control). Blood samples from 56 mild asthmatics, 67 severe asthmatics and 48 COPD patients were collected at baseline and DNA was extracted for investigation of DNA methylation status and genetic variation in *NPSR1* (Table 1).

#### BAMSE

The BAMSE (Swedish abbreviation for Children Allergy Environment Stockholm Epidemiology) study is a Swedish prospective birth cohort [23] with 4,089 infants recruited between 1994 and 1996. Data related to environmental exposures and symptoms of asthma and allergic diseases in the children have been obtained by questionnaires sent to the parents at different occasions. Peripheral blood samples drawn at 8 years of age were used for analysing serum IgE antibodies to inhalant allergens using Phadiatop (Phadia AB, Uppsala, Sweden) as well as for the current study on DNA methylation.

All children in BAMSE with a doctor's diagnosis of asthma (ever) up to 8 years of age as reported by the parents, with DNA available for analyses were selected as cases (n = 273) and children with no history of asthma or other allergic diseases with DNA available for analyses were selected as controls (n = 273) [51]. Cases and controls were obtained after exclusion of individuals with non-successful DNA methylation analyses (n = 19) resulting in 273 cases and 273 controls (Table 3). The pilot study testing the assays in human peripheral blood derived DNA was based on a random selection of all the 273 children in BAMSE with a doctor's diagnosis of asthma (ever) up to 8 years of age (n = 136) and a subsequent random selection of the 273 healthy children (n = 124). For the in depth investigation of DNA methylation status, a selection of CpG sites (number 1 to 5) were studied in relation to asthma phenotypes and the effect of exposure to environmental factors.

Four asthma phenotypes were investigated in relation to DNA methylation status; 1) asthma up to 8 years of age (doctor's diagnosed reported by the parents), 2) allergic asthma (defined as doctor's diagnosis of asthma up to 8 years in combination with an IgE-value for Phadiatop ≥0.35 kU/l; a mixture of cat, dog, horse, birch, timothy, mugwort, *Dermatophagoides pteronyssinus*, and *Cladosporium* allergens), 3) non-allergic asthma (defined as doctor's diagnosis of asthma up to 8 years in combination with an IgE-value for Phadiatop ≤0.35 kU/l), and 4) current asthma at 8 years (defined as doctor's diagnosis of asthma up to 8 years in combination with at least one episode of wheeze in the last 12 months). In addition, association between sensitization (IgE-value for Phadiatop ≥0.35 kU/l) and methylation levels were also analyzed.

Exposure to environmental tobacco smoke was classified as parental tobacco smoke exposure in infancy (daily parental smoking at birth of the child, yes/no) and current parental tobacco smoke exposure (daily parental tobacco smoke exposure at child age 8 years, yes/no). Information on air pollution exposure from road traffic (NO_x_) at residential, day care, and school addresses was estimated using dispersion modelling, and has been described elsewhere [52]. The mean level of traffic-NOx to which the child was exposed during infancy was 19.5 µg/m^3^ (median: 15.0 µg/m^3^ and 5th–95th percentile: 3.2-48.9 µg/m^3^) and current mean exposure level at 8 years of age was 9.6 µg/m^3^ (median: 7.3 µg/m^3^ and 5th–95th percentile: 1.7–27.7 µg/m^3^) in the study population (cases and controls combined). Associations were investigated for the 5th–95th percentile difference in NO_x_ exposure levels corresponding to 45.7 µg/m^3^ during first year of life (here defined as NO_x_ from traffic during infancy), and 26.0 µg/m^3^ between 7 and 8 years of age (here defined as current NO_x_ from traffic). Current dietary folate intake (energy adjusted intake at 8 years of age, in µg per day) was divided into quartiles (Q1 = 142.6 to <192.7 µg per day, Q2 = 192.7 to <212.6, Q3 = 212.6 to <235.3 µg per day, Q4 = 235.3 to 401.6 µg per day) before investigation. Current BMI (Body mass index at 8 years of age) was measured in kg/m^2^. Sampling season is the time of the year when blood samples were collected at 8 years of age, and were investigated in three-month seasons using January to March as reference category.

### Electrophoretic Mobility Shift Assay (EMSA)

Nuclear extracts from Colo205 (colon adenocarcinoma, ATCC CCL-222) and HEK293 (human embryonic kidney cells, ATCC CRL-1573) cells were prepared according to standard protocols [53]. Duplex probes encompassing the four sites CpG site 2 coinciding with rs2168890, CpG site 3, CpG site 8 coinciding with rs2530547, and CpG site 9 coinciding with rs887020 in the predicted *NPSR1* promoter (Figure 1) were produced by annealing oligonucleotide pairs specific for each allele at the polymorphic site (2.5 µmol/l, probe sequences available upon request). The four sites were chosen according to the central location of the CpG site in relation to binding of the transcription factor and the value of the transcription factor itself in relation to *NPSR1* and/or asthma. Binding reactions were performed in 25 mM HEPES pH 7.9, 150 mM KCl, 10% glycerol and 5 mM dithiothreitol, with 3 µg nuclear extract, approximately 0.5 ng [α-32P] Klenow-labeled probe and 3 µg poly dI-dC in a total volume of 20 µL. Polyacrylamide (6%) gels were run in 0.5× tris-borate-EDTA at 4°C for 3 h at 300 V, dried, and autoradiographed to visualize DNA protein complexes. Experiments were repeated three times for each nuclear cell extract (Colo205 and HEK293). Both cell extracts gave similar results and data shown is a representative gel from the Colo205 analyses.

### Isolated cell types from blood

Peripheral blood, 450 ml, was collected from six healthy adult male blood donors, age 38±13.6 years as described elsewhere [25]. Briefly, whole blood, peripheral blood mononuclear cells (PBMC), CD4^+^ T cells, CD8^+^ T-cells, CD56^+^ NK cells, CD19^+^ B cells, CD14^+^ monocytes, granulocytes, neutrophils and eosinophils were isolated. PBMC and granulocytes were isolated using Ficoll-Paque Plus™ (GE Healthcare, Sweden). Magnetic-activated cell sorting (MACS, Miltenyi Biotech, Germany) was used to obtain T cells, B cells, monocytes, and NK cells from the PBMC:s and neutrophils and eosinophils from the granulocyte containing pellet. Cell purities were controlled by fluorescence activated cell sorting (FACS) and ranged from lowest 81.8±18.8% for eosinophils to highest 97.8±1.7% for neutrophils. DNA was extracted from cells using QIAmp DNA Micro Kit (QIAGEN, Germany). 500 ng DNA was bisulfite treated using EZ-96 DNA Methylation Kit (Zymo Research Corporation, USA) according to the manufacturer's instructions, and analyzed on the Illumina Infinium 450K Bead array.

### SNP genotyping

Primers for multiplex PCR and extension reactions were designed by the SpectroDesigner software (Sequenom GmbH, San Diego, CA, USA). All primer sequences are available on request. PCR and extension reactions were performed according to the manufacturer's standard protocols. The SNP analysis in the BAMSE samples was performed by MALDI-TOF mass spectrometry (matrix-assisted laser desorption/ionisation-time of flight; Sequenom GmbH). Each assay was validated using a set of 14 trios families, in total 42 individuals. Genotype data from these individuals are available through the HapMap consortium (http://hapmap.ncbi.nlm.nih.gov/). Concordance analyses with the HapMap data as well as analysis of the parent-offspring-compatibility with the produced genotypes were performed. No significant deviation from Hardy-Weinberg equilibrium (P>0.05 using χ^2^ test) was seen for any of the SNPs and the average genotyping success rate was >95%. BIOAIR samples were genotyped using TaqMan allelic discrimination on the ABI Prism 7500 detection system according to the manufacturer's protocol (Assays used: C__16245540_10, C__16245541_10, C___2959938_10, and C__2959937_10, Applied Biosystems, CA, USA). Reruns of a small set of samples for all four genotypes showed 100% success rate.

### DNA methylation

The promoter region [24] used for primer design contained 12 CpG sites and four of these were affected by polymorphisms (rs2168891 affecting CpG site 1, rs2168890 affecting CpG site 2, rs2530547 affecting CpG site 8 and rs887020 affecting CpG site 9, Figure 1). As a quality check for the methylation pattern in the promoter of *NPSR1*, a second region with a predicted CTCF binding site which is of interest for DNA methylation was chosen for analysis in the intron 4 region. This region constituted 1053 bp (NCBI36, chr7:34829940–34830992) and contained 11 CpG sites (Figure S2). Two sites, CpG site 2 and 5, were affected by polymorphism (rs17199818 and rs17199853, respectively). Primers for the regions were designed using EpiDesigner (www.epidesigner.com, Sequenom, CA, USA, Table S2). One µg of DNA obtained from peripheral blood cells was modified by sodium bisulfite using the EZ DNA Methylation™ Kit (Zymo Research, CA, USA). Sodium bisulfite DNA was analysed using EpiTYPER according to manufacturer's recommendations & protocol [26]. All EpiTYPER designs were quality tested by analysing standard curves with methylated and unmethylated DNA mixed in ratios resulting in 0%, 25%, 50%, 75%, and 100% methylated DNA. This information resulted in the exclusion of CpG site 7 in the promoter region and CpG site 5 in the CTCF binding region from further analyses due to poor estimation of DNA methylation (Figure S3). All samples were distributed randomly on all plates based on disease phenotype, samples were analysed in duplicates and samples with a standard deviation above 0.3 were removed from further analysis. Two CpG sites (CpG site 5 and 8 in the promoter) were included in two separate designs and showed similar methylation levels for both assays (Table S1). A quick analysis of the intron 4 region revealed a similar pattern of DNA methylation compared to the promoter region. No differences in DNA methylation based on respiratory phenotype were found and therefore, this region was not studied further.

### Statistical analyses

None of the continuous variables deviated from normal distribution including DNA methylation levels (Figure S4). For basic comparisons of clinical characteristics, DNA methylation in relation to asthma and other obstructive airway disease and genotype effects on DNA methylation, Analysis of variance (ANOVA) when considering three groups or Student's T-test when considering two groups were used. Distribution regarding genotype and gender was compared using chi square test.

For the investigation of promoter methylation status and asthma and other obstructive airway disease in the larger selection of samples from the BIOAIR study (n = 171) and the BAMSE cohort (n = 546), crude and adjusted linear regression model was used (methylation levels as a continuous outcome). In the BIOAIR analyses, the linear regression models were adjusted for the potential confounders gender, age and country of origin for all variables analysed. Linear regression analyses performed for the BAMSE data were adjusted for the potential confounders gender, age at blood sampling and plate used for sodium-bisulfite treatment. For the investigation of DNA methylation status and the effect of exposure to environmental factors linear regression models were used adjusting for gender, age at blood sampling and plate used for sodium-bisulfite treatment. For analyses of air pollution effects, adjustment for municipality was also done. In addition, adjustment for all the other exposure variables analyzed in Table 5 did not change the results; neither did adjustment for doctor's diagnosis of asthma (ever) up to 8 years of age. STATA (StataCorp LP, TX, USA) was used for all statistical analyses.

## Supporting Information

Figure S1 The levels of DNA methylation (%) for each CpG site studied in relation to age in years (dot blots, left column of graphs) or material (box plots, right column of graphs). Dots with green color in the box plot defines mild outliers (values that are under three interquartile range (IQR) from the 25th and 75th percentiles) and red dots defines severe outliers (values that are outside of three IQR:s from the 25th and 75th percentiles). BAMSE - Swedish abbreviation for Children Allergy Environment Stockholm Epidemiology, BIOAIR - the BIOmarkers of severe chronic AIRways disease.
(PDF)

Figure S2 The genetic sequence for the regions defined to bind CTCF in intron 4 of Neuropeptide S Receptor 1 (*NPSR1*) gene. Yellow regions indicate the CpG sites and red nucleotides indicate genetic variants.
(PDF)

Figure S3 Standard curves obtained from the EpiTYPER analysis of bisulfite treated samples. We designed three assays in order to cover the Neuropeptide S Receptor 1 (*NPSR1*) gene promoter region. CpG site 5 and CpG site 8 are covered by two designs. CpG site 7 failed the validation and was excluded from analysis.
(PDF)

Figure S4 The CpG sites analyzed showed normal distribution. Data is displayed via histogram plots in the left column of graphs and via probability plots in the right column of graphs.
(PDF)

Figure S5 DNA methylation levels (%) in the *NPSR1* promoter region in blood cells from six healthy adult male blood donors (age 38±13.6 years). DNA methylation was analyzed using EpiTYPER and the designs provided (Table S2).
(PDF)

Table S1 DNA methylation (%) in the Neuropeptide S Receptor 1 (NPSR1) gene analyzed using EpiTYPER in two two well characterized cohorts that offer unique opportunities to investigate genetics and epigenetics in relation to respiratory disease phenotypes and environmental exposures; the BIOAIR study of severe asthma in adults and the Swedish birth cohort BAMSE.
(PDF)

Table S2 EpiTYPER primer sequences for the Neuropeptide S receptor 1 (NPSR1) gene.
(PDF)
