# Supplementary figures and images for: DNA Methylation in the Neuropeptide S Receptor 1 (NPSR1) Promoter in Relation to Asthma and Environmental Factors
Source: PLoS One. 2013 Jan 23;8(1):e53877. doi: 10.1371/journal.pone.0053877 (PMC3553086; doi:10.1371/journal.pone.0053877)

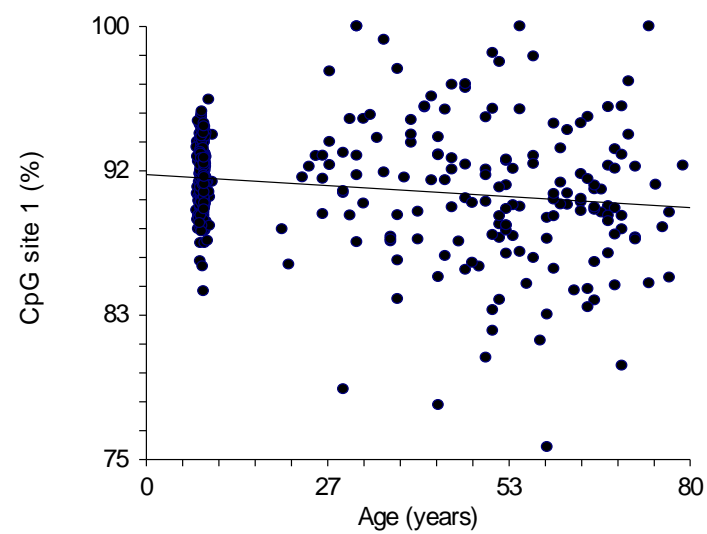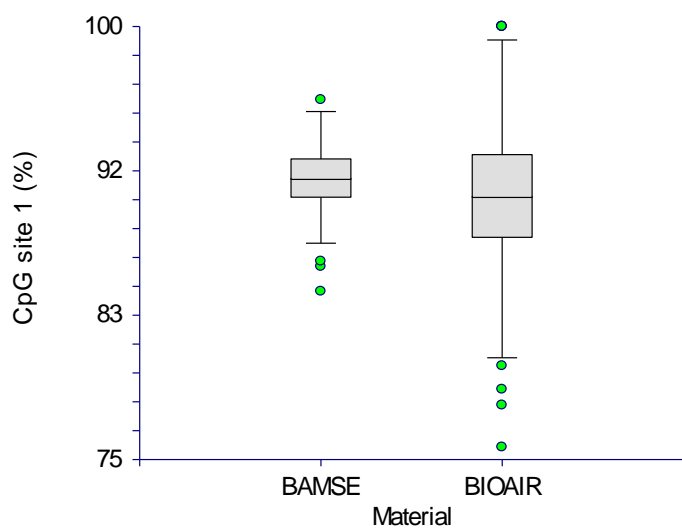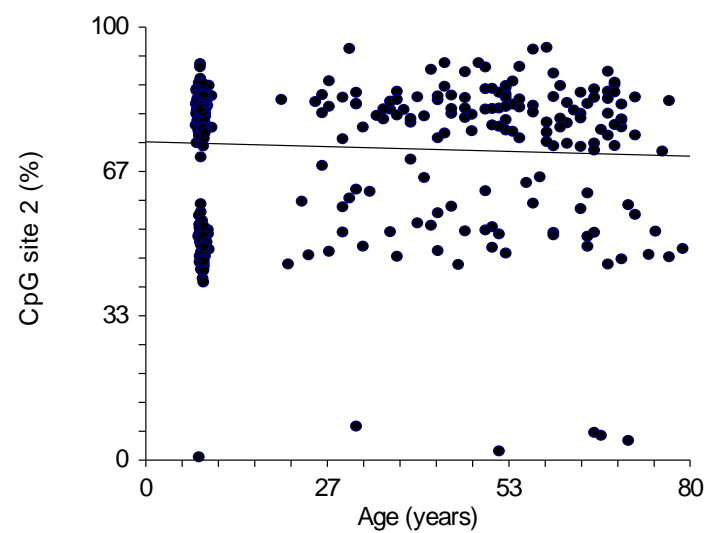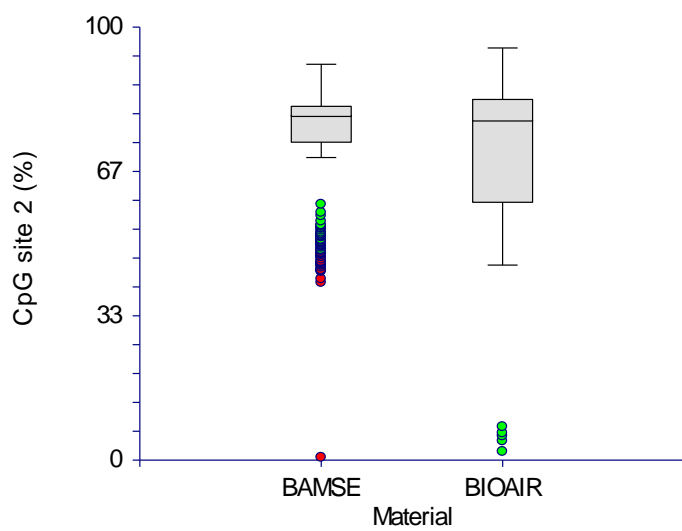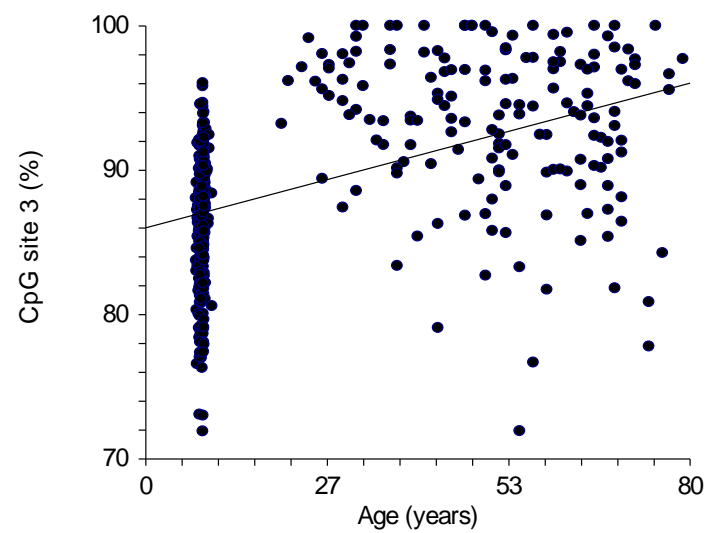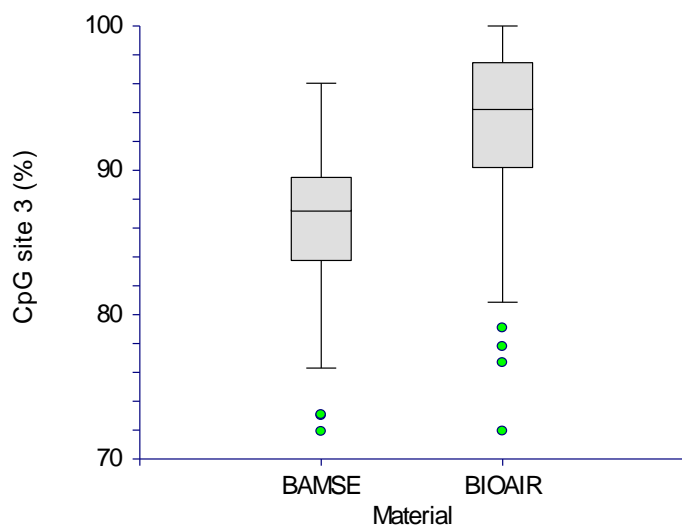

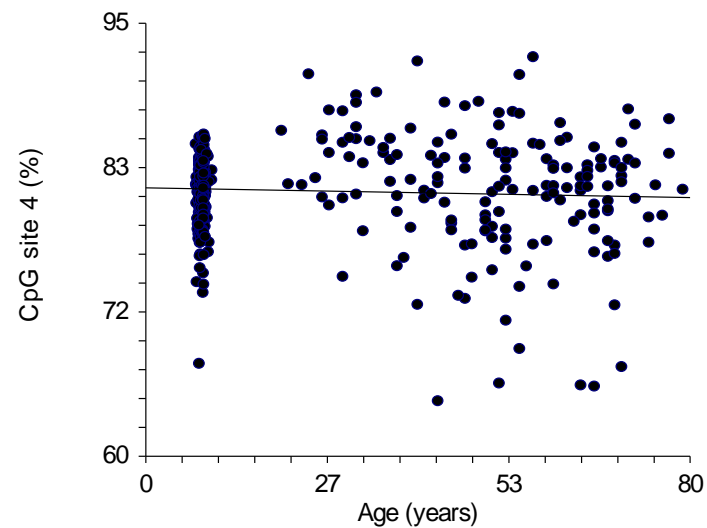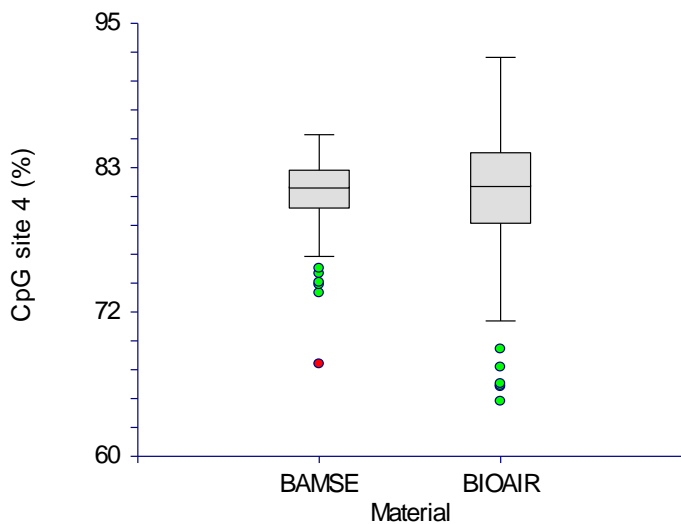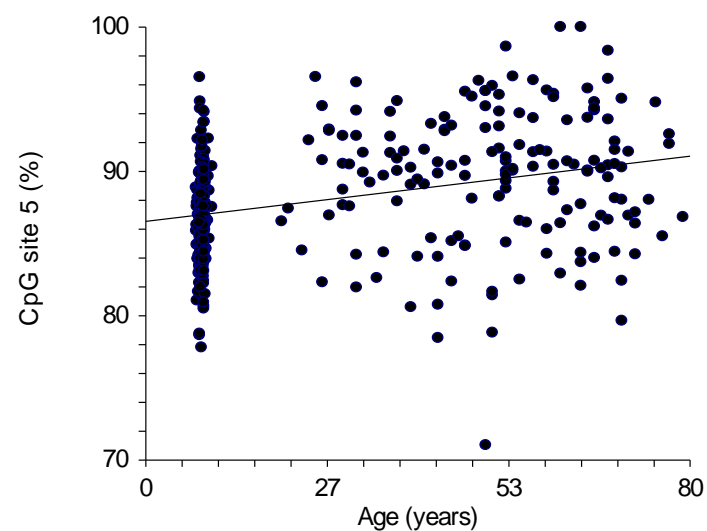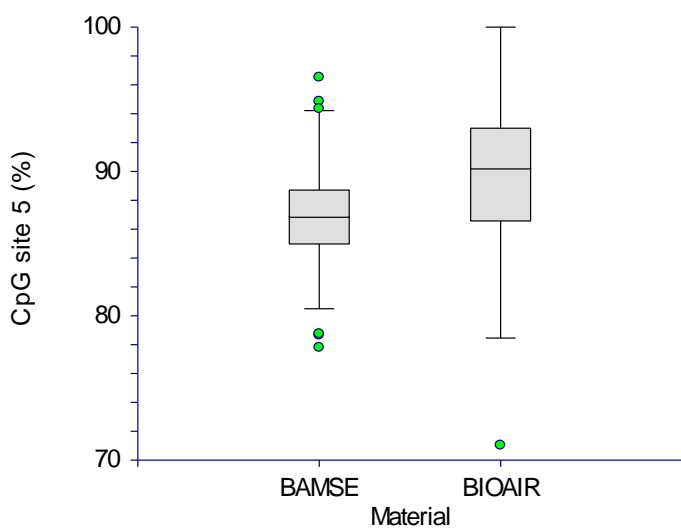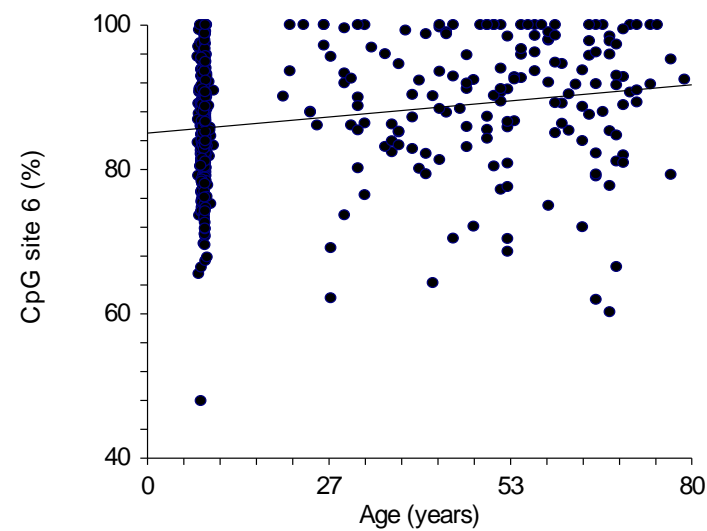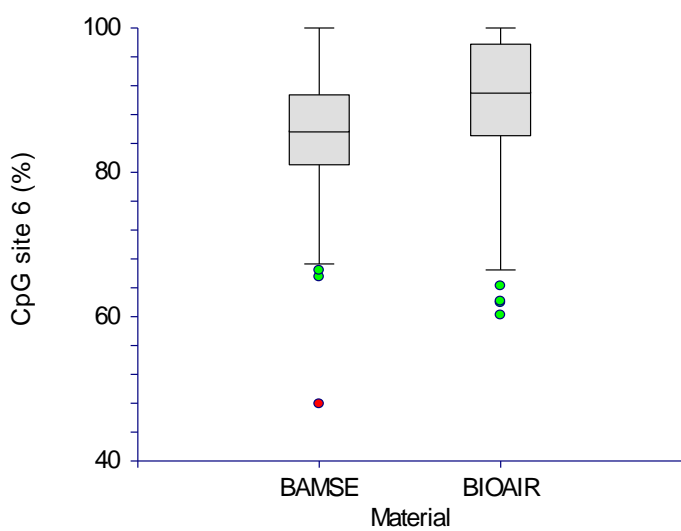

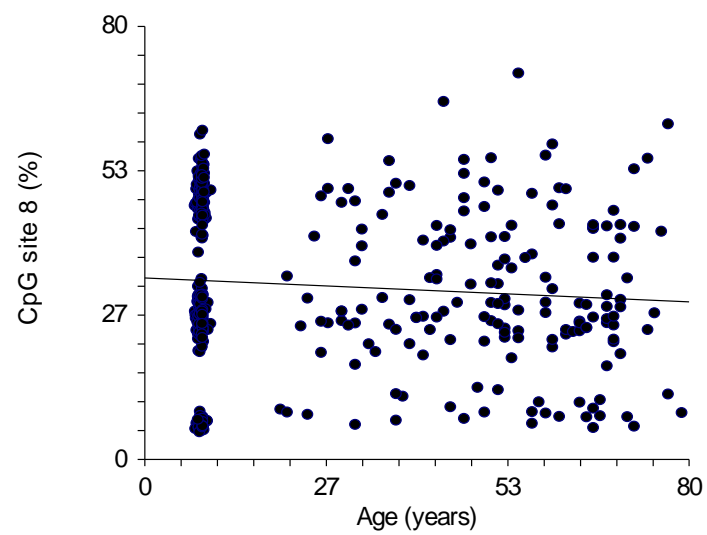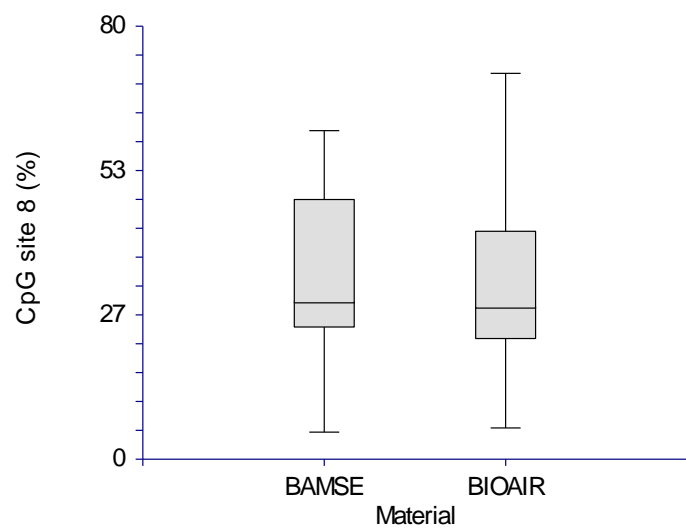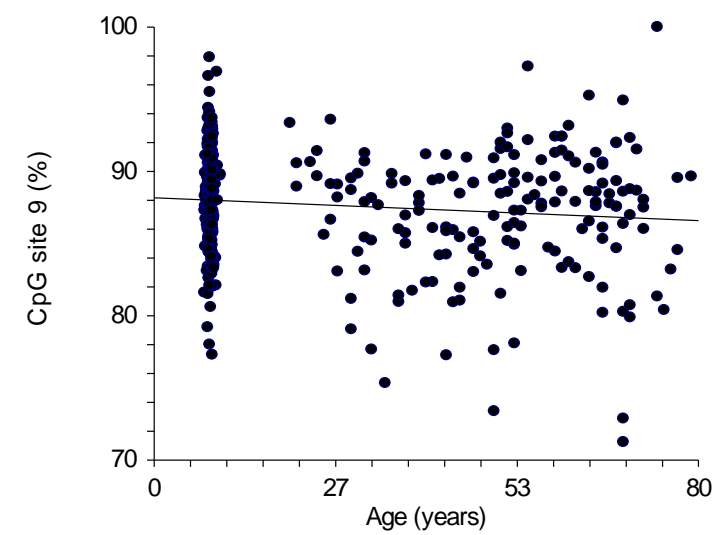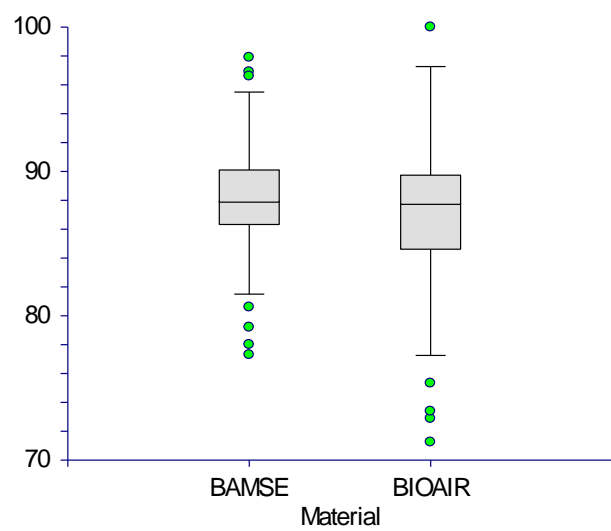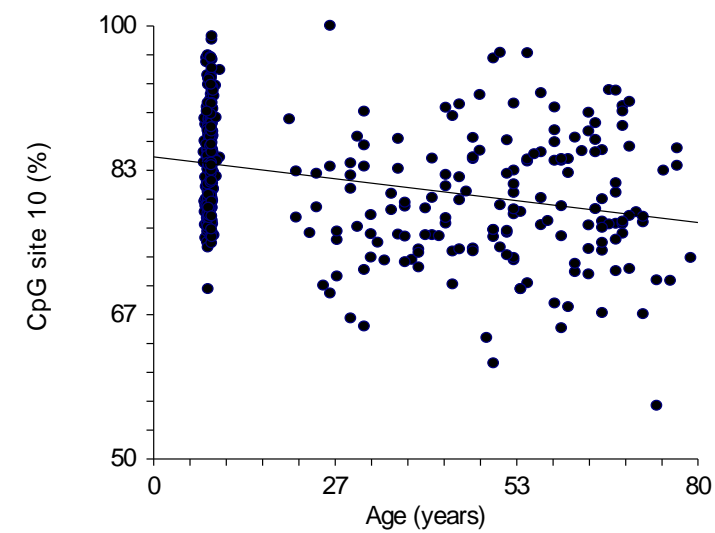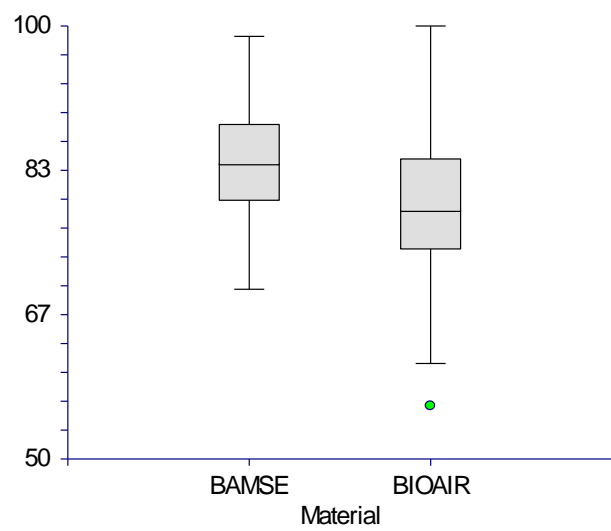

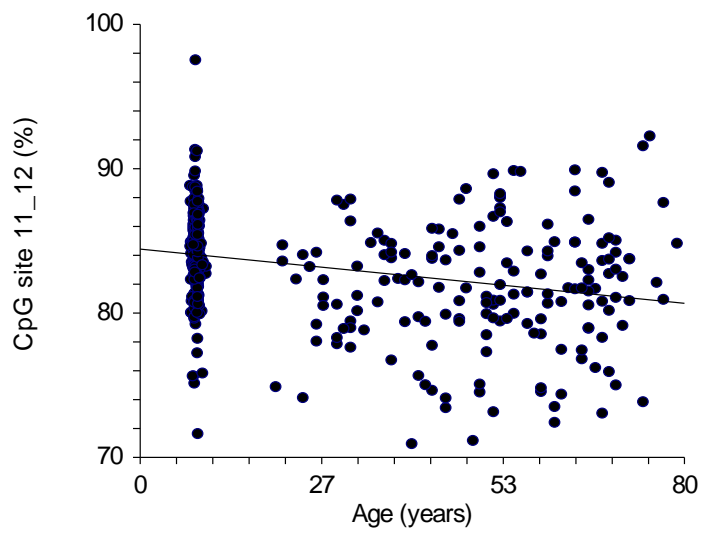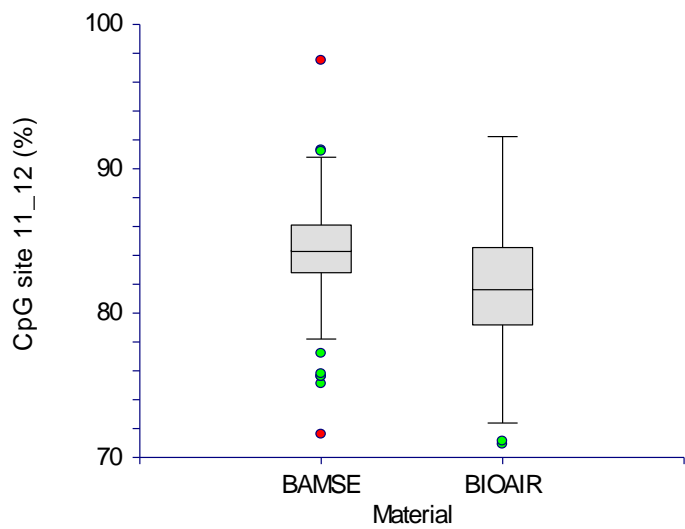

Supplement: Figure S1 — The levels of DNA methylation (%) for each CpG site studied in relation to age in years (dot blots, left column of graphs) or material (box plots, right column of graphs). Dots with green color in the box plot defines mild outliers (values that are under three interquartile range (IQR) from the 25th and 75th percentiles) and red dots defines severe outliers (values that are outside of three IQR:s from the 25th and 75th percentiles). BAMSE - Swedish abbreviation for Children Allergy Environment Stockholm Epidemiology, BIOAIR - the BIOmarkers of severe chronic AIRways disease. (PDF) [file pone.0053877.s001.pdf]

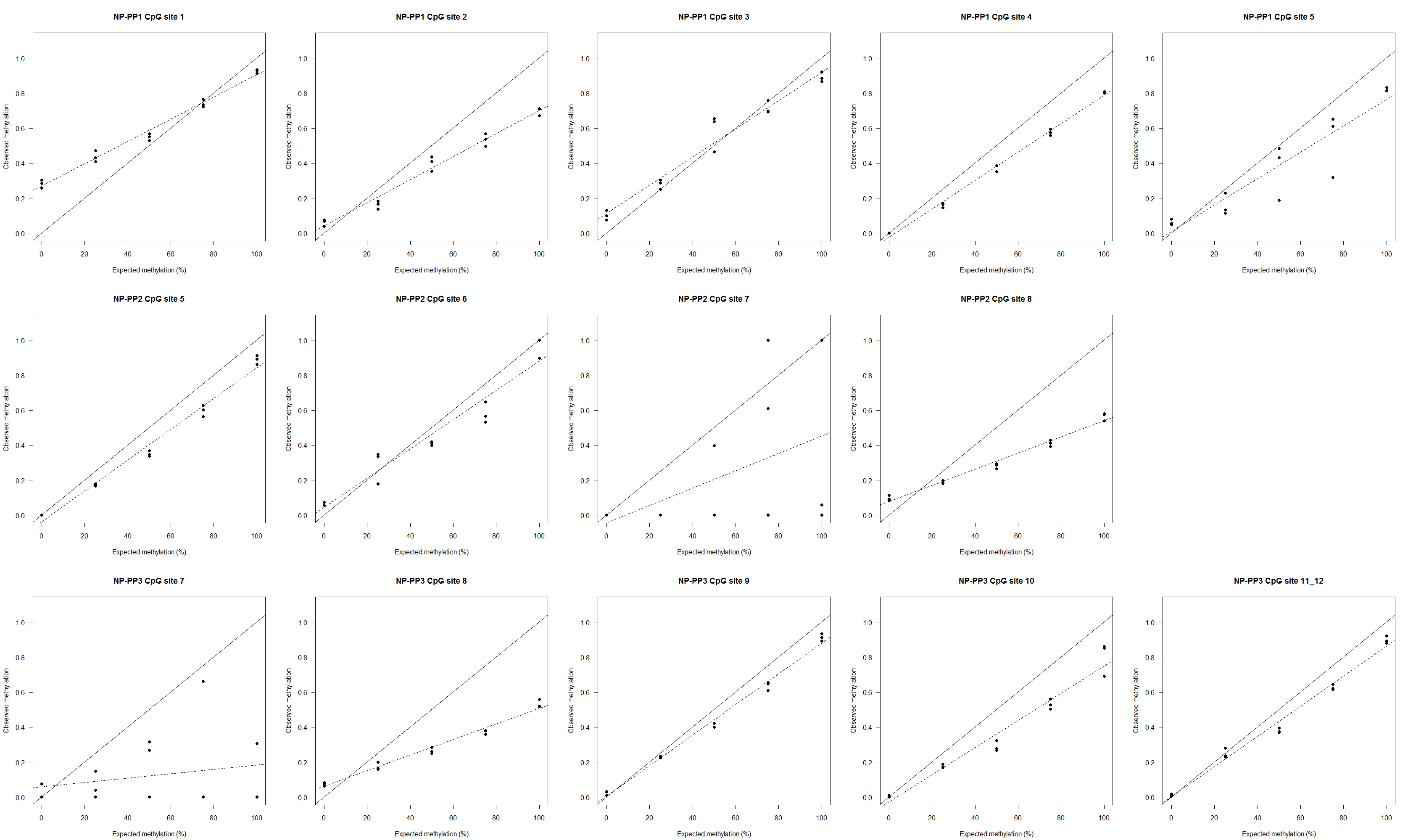

Supplement: Figure S3 — Standard curves obtained from the EpiTYPER analysis of bisulfite treated samples. We designed three assays in order to cover the Neuropeptide S Receptor 1 (NPSR1) gene promoter region. CpG site 5 and CpG site 8 are covered by two designs. CpG site 7 failed the validation and was excluded from analysis. (PDF) [file pone.0053877.s003.pdf]

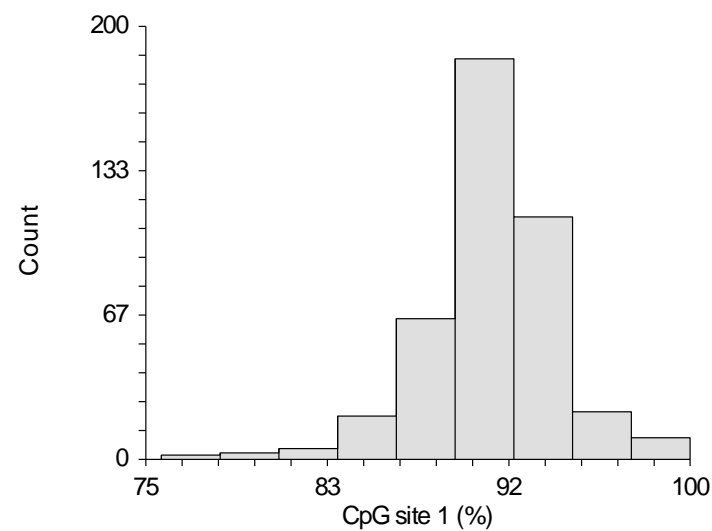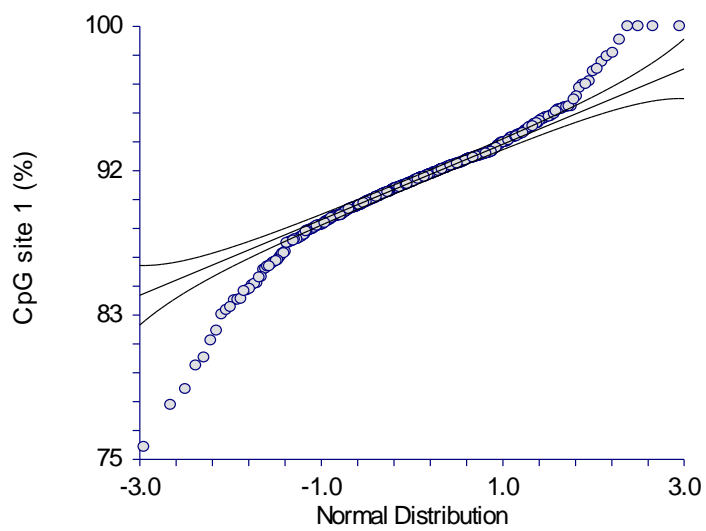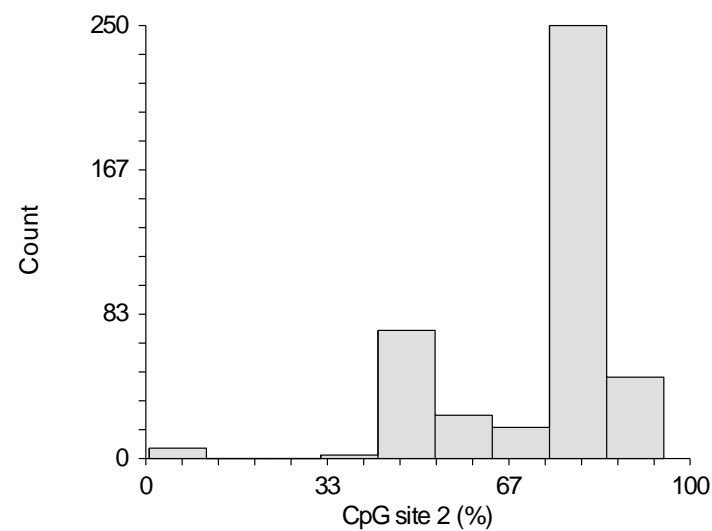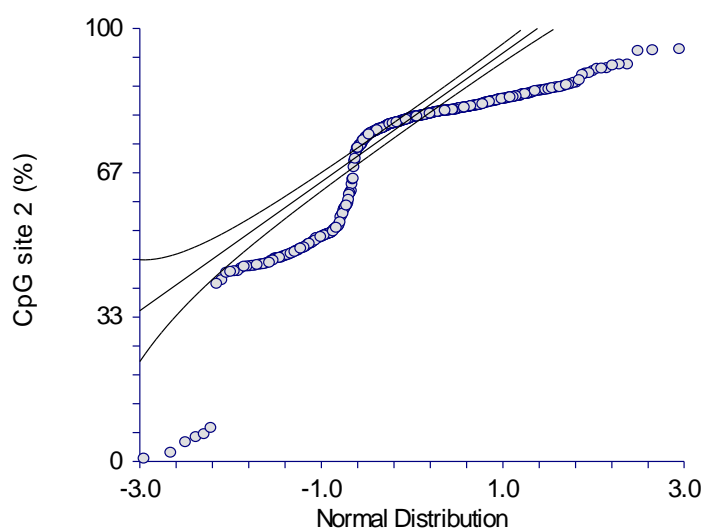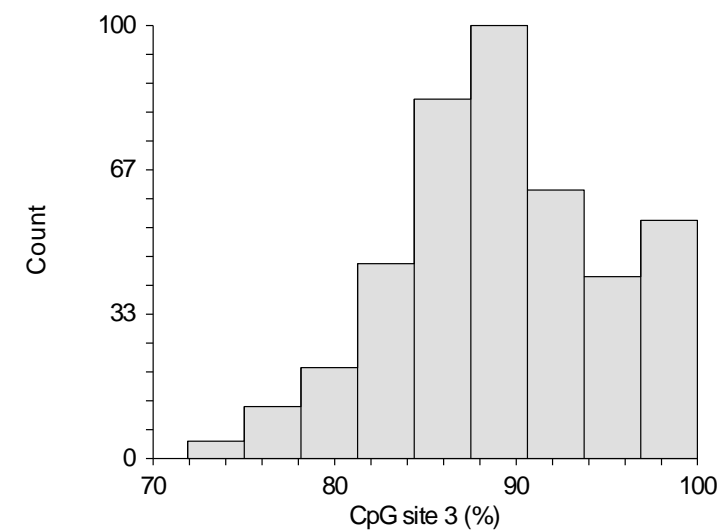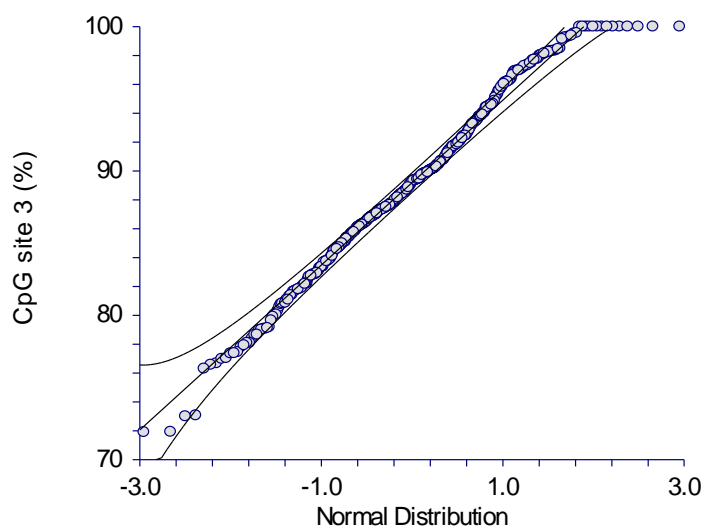

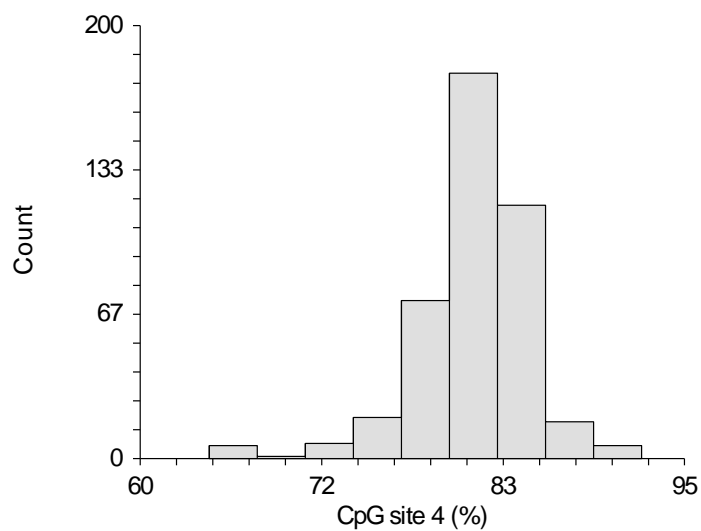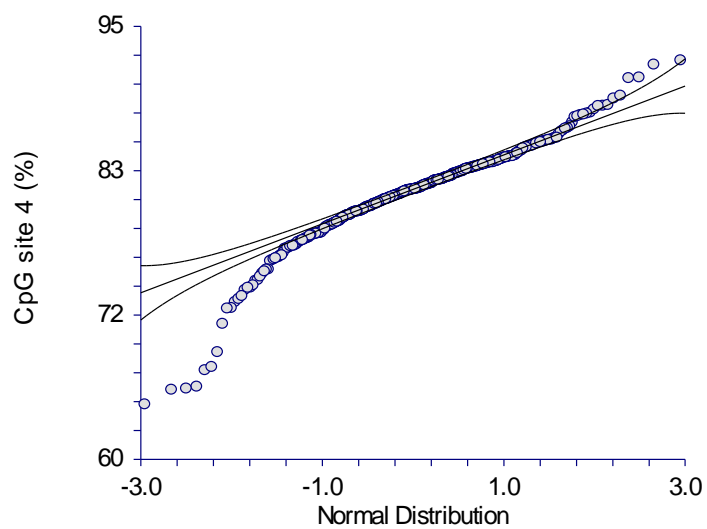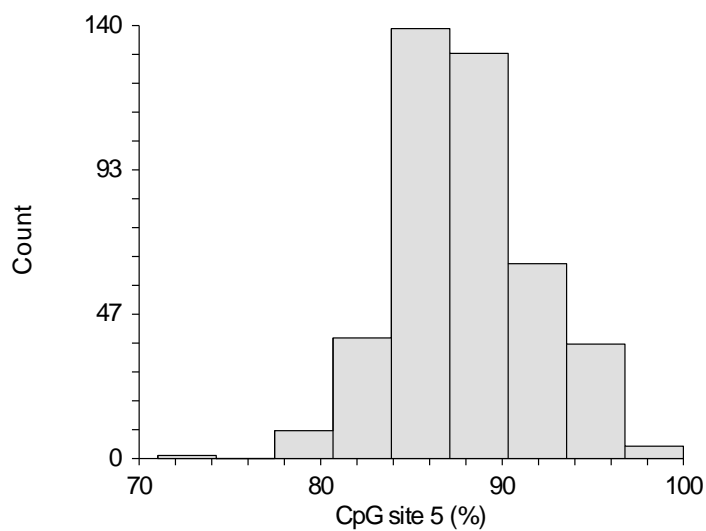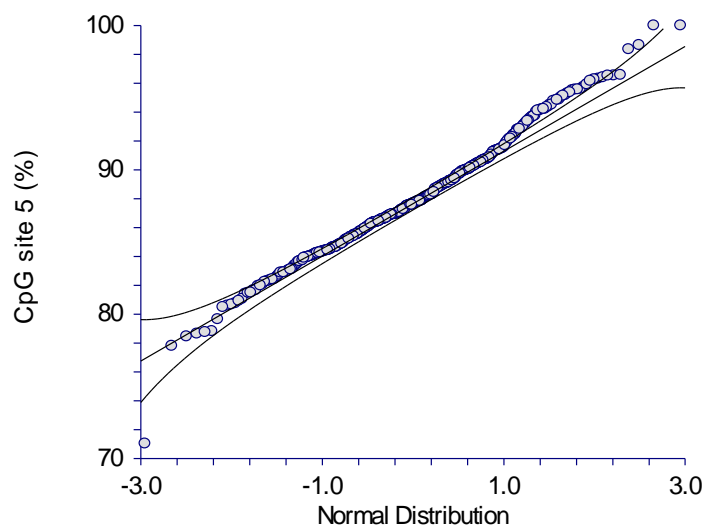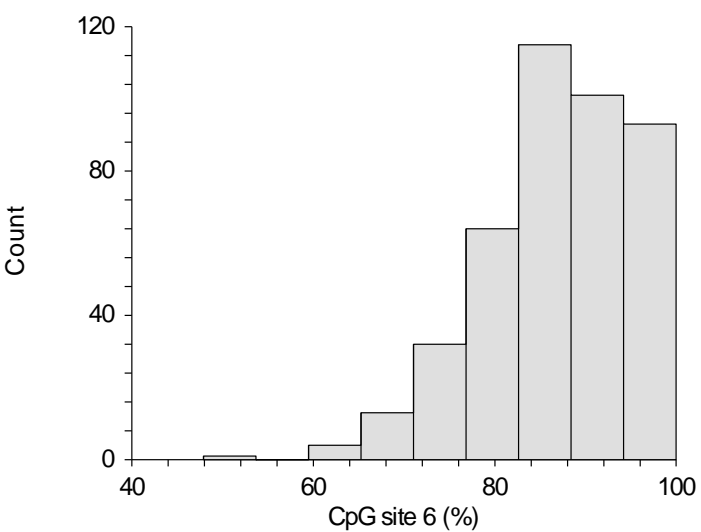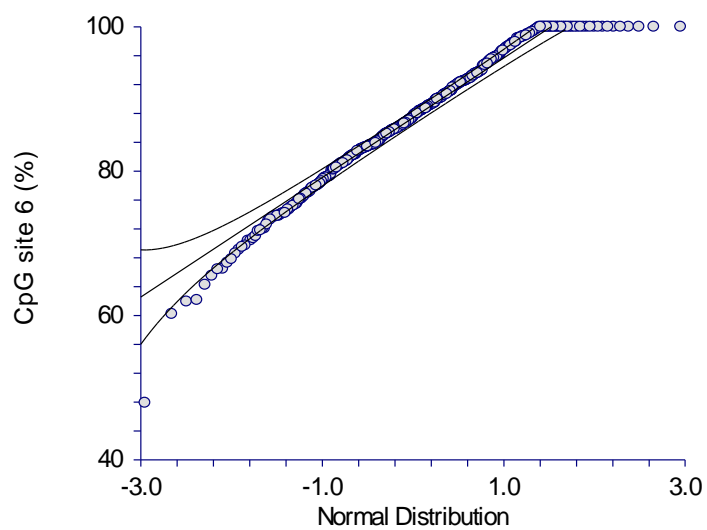

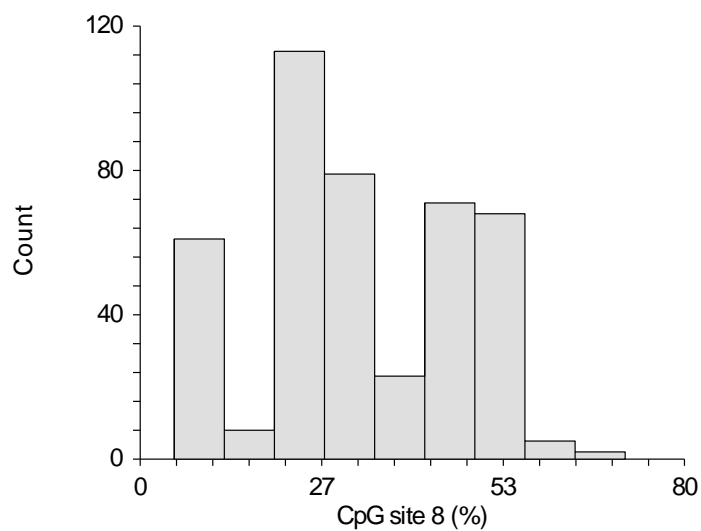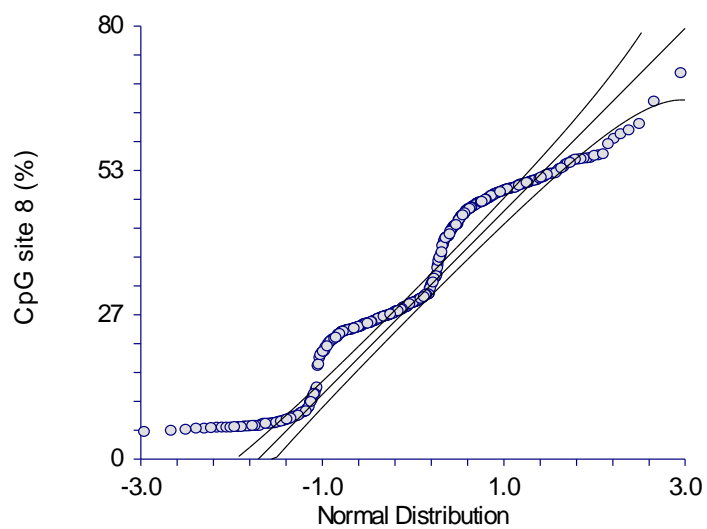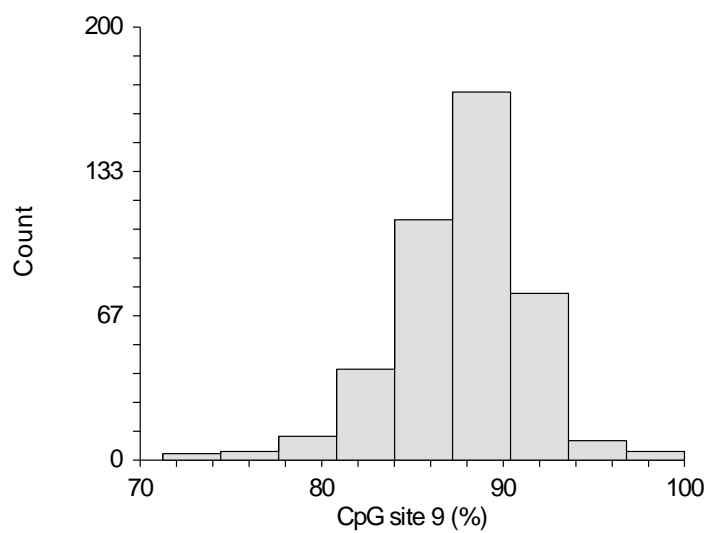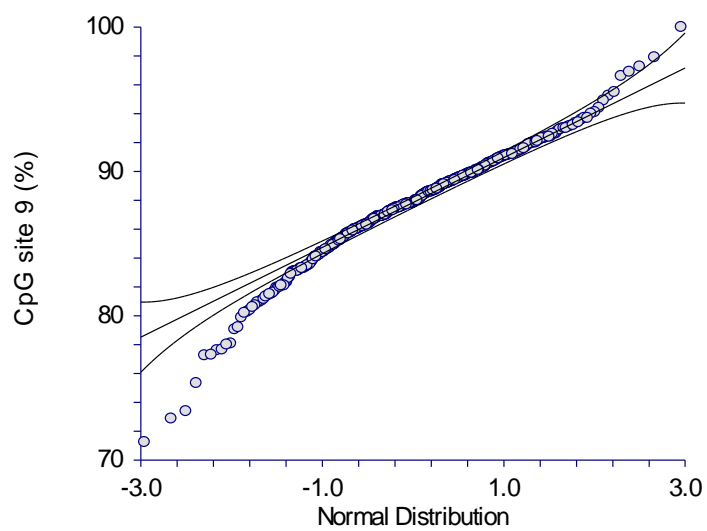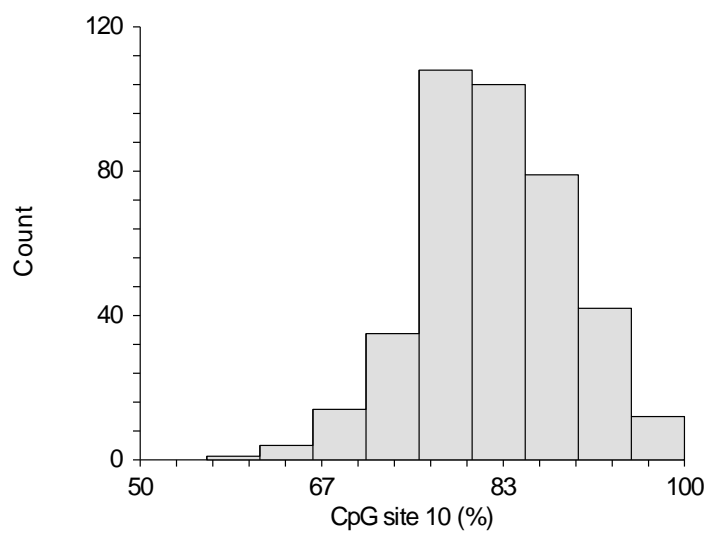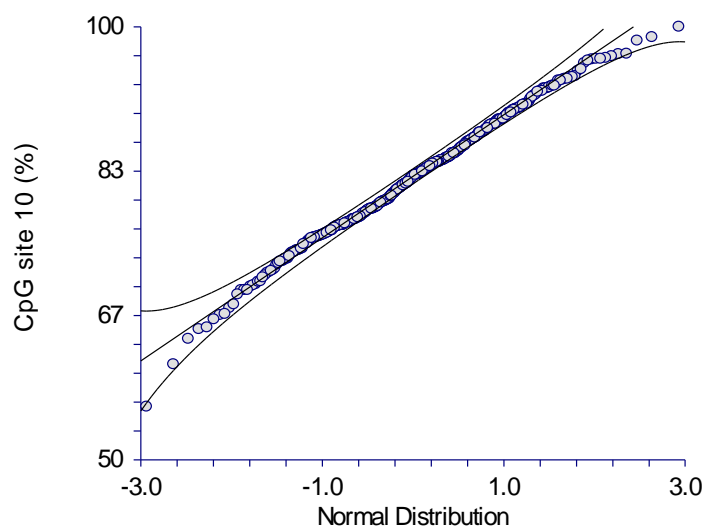

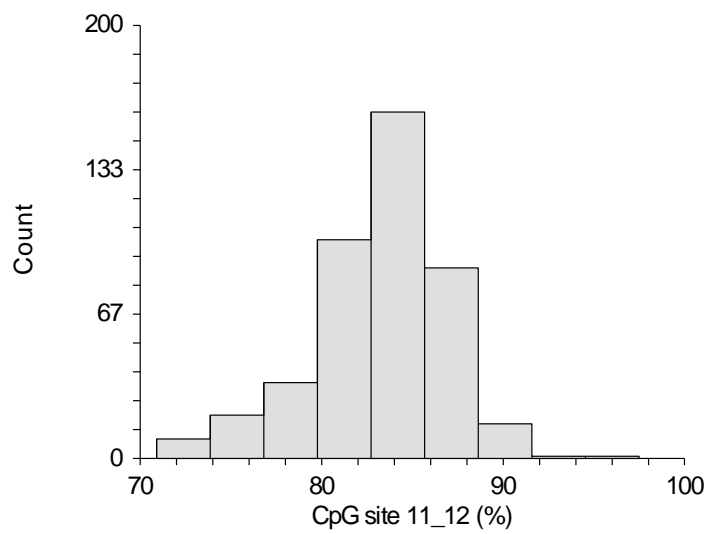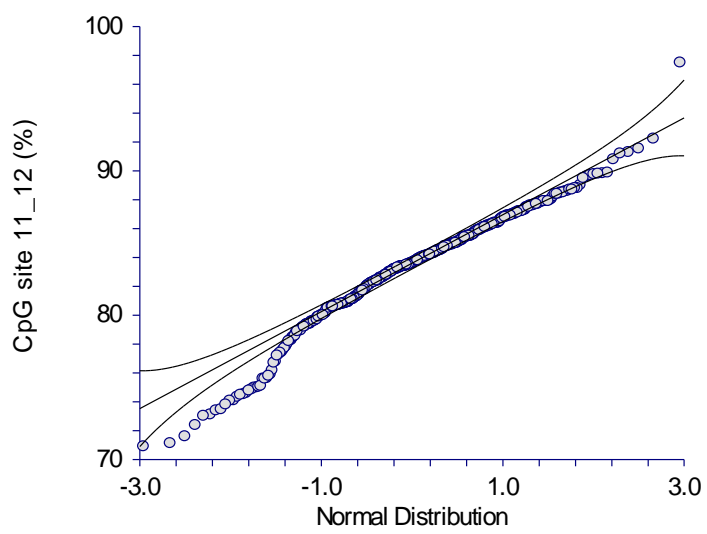

Supplement: Figure S4 — The CpG sites analyzed showed normal distribution. Data is displayed via histogram plots in the left column of graphs and via probability plots in the right column of graphs. (PDF) [file pone.0053877.s004.pdf]

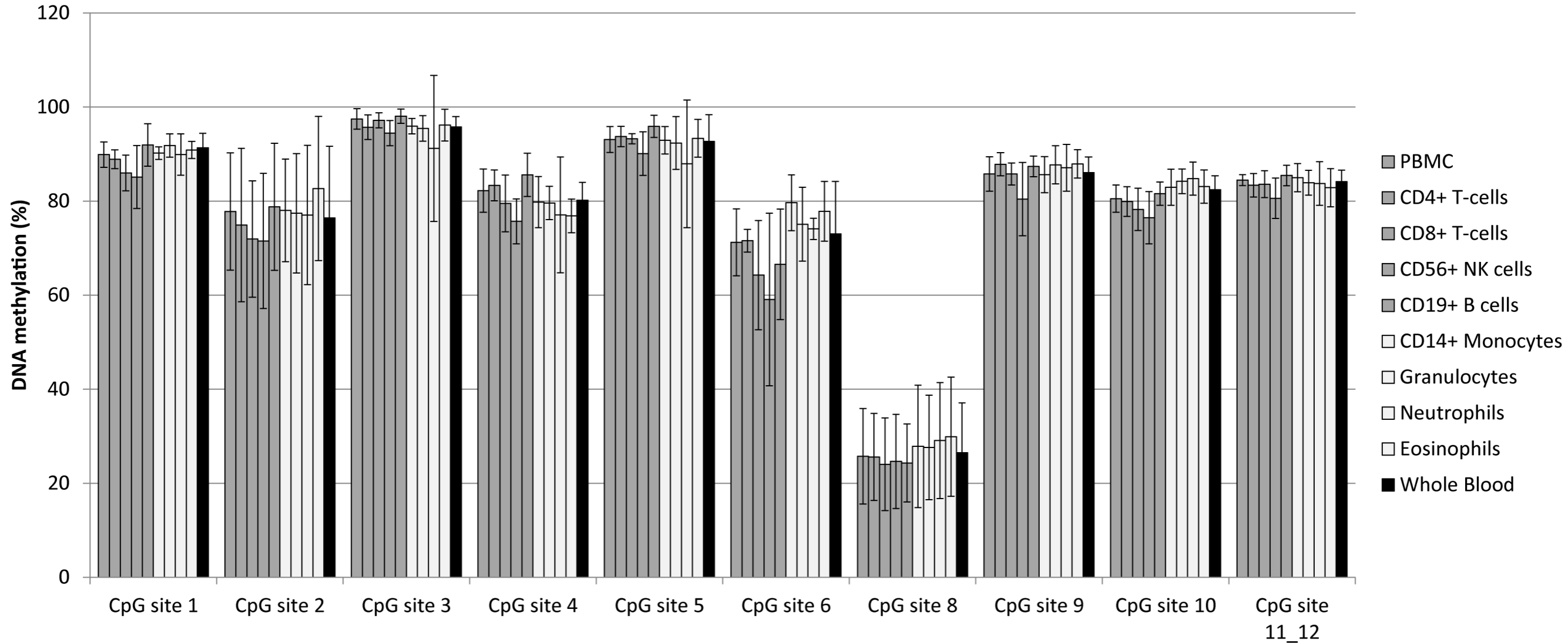

Supplement: Figure S5 — DNA methylation levels (%) in the NPSR1 promoter region in blood cells from six healthy adult male blood donors (age 38±13.6 years). DNA methylation was analyzed using EpiTYPER and the designs provided (Table S2). (PDF) [file pone.0053877.s005.pdf]
