# Supplementary material for: DNA Methylation in the Neuropeptide S Receptor 1 (NPSR1) Promoter in Relation to Asthma and Environmental Factors
Source: PLoS One. 2013 Jan 23;8(1):e53877. doi: 10.1371/journal.pone.0053877 (PMC3553086; doi:10.1371/journal.pone.0053877)
Supplement: Figure S2 — The genetic sequence for the regions defined to bind CTCF in intron 4 of Neuropeptide S Receptor 1 (NPSR1) gene. Yellow regions indicate the CpG sites and red nucleotides indicate genetic variants. (PDF) [file pone.0053877.s002.pdf]

> **CTCF peak:** dna:chromosome 7:NCBI36:7:34829940:34830992:Forward
GTTATTTATCAGTGCTTTTCCAATTGCACTTCCCACAGTGACCCCCAGAGGGCTCATTTT
GCTGGCCTGCTCCAGGGCAGATCTGGGAGCTGCTGGCTGTCTCAGTTTCAACCAAAGATA
CCTGAGCTTTCAACACTTTCCTATACTGGGTTCGAGATAAAATTATATTTGAAGAAAGGA
TCTTACTACCTGAAAAAAAATGCCACTTGTACAAGTTCATATTTCTTAACAGGAAAAGAT
GGTCATAACAGGCTTTTCAGAGGAAAAAAATTAG**N**TTACAA**N**GTAGGATCTGTATCATGA
TCTCATTTCTAAATGTGTAATCTATGTATTATATATGCATG**C**GTTATCAAAATTTAAGCT
TGACGTTTCTGGTTGGTGCCACTATGGATTTTTTT**N**CTTTTCGCTTATTTTTATTTGCTA
GTGTTTCTATAATCAATGTGTGTAACTTATA**C**GATTTTATAAAAAGGTAAATGAGTTACA
TGGAAAAGAGGAAGGCTAAGCGGCAGAGAAGTACAGAAATGGACAAGAGCACATGGAAGA
GGGAGTCTGACTTTCCGGGGGTGAGAAACATGAGACAGTCTACCTTATATGCAAACCTGC
ATTTCATCTGTTCGATCCTGGATCCTTCAGCCCTGAGAAGTCACTCTTCATGGCAAATGG
GCTCAGCTGCCTGGAAGGCCATCAGTCTCCCTGCTGTGGCAGCTGCGTCACAGCCTAAGA
GAGTGGGGCTTTCACAGTGTAGCCCCAGCACCAGTAAGAGAAAAACAGCTCCAAGCCTAT
ACCCTGCTGGGCCTTTGGGGAAACATCTTCGGAGACCTTGGTAGAATTGATTCTAAAGTA
TGATTCTATACTCTCTTCCCTGTCCATCCCTCTCAAAAGTGAATGCACTGTTCATCCATC
AAGTGGAAACTTTGTGGGGCCCAG**N**AGTCACACGTTGCCCTTAAGAGTGCCCTGGAACCT
ATAGAAGAAGATGACATTAGAATTCTAACCATTGAAAGAACAAGAGCACAGGGGGCAGCT
ACT**N**ATGTCACCCAGGACCATAGAAACCCATCA
