# Supplementary material for: DNA Methylation in the Neuropeptide S Receptor 1 (NPSR1) Promoter in Relation to Asthma and Environmental Factors
Source: PLoS One. 2013 Jan 23;8(1):e53877. doi: 10.1371/journal.pone.0053877 (PMC3553086; doi:10.1371/journal.pone.0053877)
Supplement: Table S1 — DNA methylation (%) in the Neuropeptide S Receptor 1 (NPSR1) gene analyzed using EpiTYPER in two two well characterized cohorts that offer unique opportunities to investigate genetics and epigenetics in relation to respiratory disease phenotypes and environmental exposures; the BIOAIR study of severe asthma in adults and the Swedish birth cohort BAMSE. (PDF) [file pone.0053877.s006.pdf]

Supplementery Table S1. DNA methylation (%) in the Neuropeptide S Receptor 1 (*NPSR1*) gene analyzed using EpiTYPER in two two well characterized cohorts that offer unique opportunities to investigate genetics and epigenetics in relation to respiratory disease phenotypes and environmental exposures; the BIOAIR study of severe asthma in adults and the Swedish birth cohort BAMSE.

	**BIOAIR**								**BAMSE**				
	**Mild Adult Asthma**		**Severe Adult Asthma**		**COPD**				**Healthy children**		**Asthmatic children**		
	**n**	**DNA methylation (%)**	**n**	**DNA methylation (%)**	**n**	**DNA methylation (%)**	**p-value**		**n**	**DNA methylation (%)**	**n**	**DNA methylation (%)**	**p-value**
NP1_CpG1*	45	89.7 ± 4.6 (75.7-99.2)	54	90.3 ± 4.1 (78.1-98.3)	42	89.9 ± 4.0 (80.4-100)	ns	NP1_CpG1*	104	91.2 ± 1.7 (84.7-94.8)	119	91.1 ± 1.6 (86.1-94.9)	ns
NP1_CpG2*	35	82.4 ± 5.9 (69.4-95.2)	43	82.0 ± 4.4 (74.3-94.8)	29	79.0 ± 6.4 (62.1-90.8)	0.05	NP1_CpG2*	92	80.6 ± 3.0 (73.1-91.4)	105	80.7 ± 3.3 (69.9-87.0)	ns
NP1_CpG3	54	94.5 ± 4.5 (81-7-100)	64	92.9 ± 5.2 (71.9-100)	46	92.8 ± 6.3 (76.7-100)	ns	NP1_CpG3	124	86.3 ± 4.4 (71.9-96.0)	136	86.7 ± 4.3 (76.3-95.8)	ns
NP1_CpG4	54	82.4 ± 4.1 (72.2-91.9)	64	81.0 ± 5.6 (64.4-92.2)	46	80.9 ± 5.4 (65.6-90.8)	ns	NP1_CpG4	124	81.5 ± 2.4 (74.1-85.8)	136	81.4 ± 2.6 (67.5-86.0)	ns
NP1_CpG5	54	91.1 ± 3.9 (84.1-100)	64	88.2 ± 4.8 (78.5-100)	45	90.0 ± 4.9 (71.0-98.4)	0.004	NP1_CpG5	124	87.1 ± 3.1 (77.8-94.8)	136	86.7 ± 2.6 (80.4-96.5)	ns
NP2_CpG5	55	81.9 ± 3.3 (73.8-87.7)	67	80.9 ± 4.1 (72.1-93.3)	48	81.2 ± 4.3 (74.0-92.2)	ns	NP2_CpG5	124	86.8 ± 2.6 (75.7-92.1)	135	87.1 ± 2.6 (75.2-92.0)	ns
NP2_CpG6	55	90.1 ± 8.3 (64.2-100)	64	89.2 ± 9.3 (62.1-100)	46	89.7 ± 10.0 (60.2-100)	ns	NP2_CpG6	123	86.0 ± 7.3 (69.7-100)	135	85.1 ± 8.4 (47.9-100)	ns
NP2_CpG7		NA		NA		NA		NP2_CpG7		NA		NA	
NP2_CpG8*	16	46.4 ± 9.7 (28.3-61.5)	27	45.2 ± 8.5 (21.0-59.9)	18	45.5 ± 5.3 (36.8-54.4)	ns	NP2_CpG8*	51	49.8 ± 4.4 (40.3-61.0)	54	50.7 ± 3.7 (42.0-57.9)	ns
NP3_CpG8*	16	45.7 ± 10.4 (26.2-66.0)	27	44.3 ± 7.2 (24.7-59.1)	18	47.9 ± 9.3 (36.9-71.3)	ns	NP3_CpG8*	51	48.9 ± 4.1 (38.2-60.0)	54	49.2 ± 3.7 (40.8-60.7)	ns
NP3_CpG9*	10	84.2 ± 3.3 (77.6-88.3)	20	84.8 ± 3.0 (79.1-89.1)	10	85.9 ± 4.1 (79.9-92.3)	ns	NP3_CpG9*	44	86.2 ± 3.1 (81.6-96.6)	37	86.6 ± 2.2 (82.1-90.3)	ns
NP3_CpG10	54	78.4 ± 6.9 (61.0-92.6)	64	80.6 ± 7.2 (65.3-100)	47	79.3 ± 8.4 (56.1-96.9)	ns	NP3_CpG10	111	84.5 ± 5.7 (69.6-98.8)	123	84.4 ± 5.6 (75.5-96.6)	ns
NP3_CpG11/12	56	81.2 ± 4.3 (71.1-88.6)	67	81.6 ± 4.3 (70.9-89.9)	48	82.4 ± 4.4 (73.0-92.2)	ns	NP3_CpG11/12	124	84.3 ± 3.2 (75.1-97.5)	136	84.3 ± 2.5 (71.6-91.3)	ns

Data are presented as mean ± standard deviation, min-max levels within parantheis. P-values are calculated using ANOVA or Students' T-test. *CpG sites coinciding with a polymorphism (NP1_CpG1 - rs2168891, NP1_CpG2 - rs2168890, NP2_CpG8 and NP3_CpG8 - rs2530547, NP3_CpG9 - rs887020). COPD - Chronic Obstructive Pulmonary Disease, NA - Not analyzed (NP2_CpG7 failed analysis), NP1 - *NPSR1* promoter design 1 for EpiTYPER, NP2 - *NPSR1* promoter design 2 for EpiTYPER, NP3 - *NPSR1* promoter design 3 for EpiTYPER. NP1_CpG5 (CpG site 5) and NP3_CpG8 (CpG site 8) were used for further analysis.
