# Supplementary material for: DNA Methylation in the Neuropeptide S Receptor 1 (NPSR1) Promoter in Relation to Asthma and Environmental Factors
Source: PLoS One. 2013 Jan 23;8(1):e53877. doi: 10.1371/journal.pone.0053877 (PMC3553086; doi:10.1371/journal.pone.0053877)
Supplement: Table S2 — EpiTYPER primer sequences for the Neuropeptide S receptor 1 (NPSR1) gene. (PDF) [file pone.0053877.s007.pdf]

Supplementary Table S2: EpiTYPER primer sequences for the Neuropeptide S receptor 1 (NPSR1) gene.

**Name**	**Sequence**
NP-PP1_F	aggaagagagGGATGAGATTTTTGTTTTGTTTTGT
NP-PP1_R	cagtaatacgactcactatagggagaaggctATAAAAATCTTCACCAAACCCCTC
NP-PP2_F	aggaagagagAGTTGGAGTAGAAGTGGGGGTTTAT
NP-PP2_R	cagtaatacgactcactatagggagaaggctAAAAATCCAACTTACCTCCTAAAAA
NP-PP3_F	aggaagagagGGTTGTGTTATGTTGGTTATTTGGT
NP-PP3_R	cagtaatacgactcactatagggagaaggctCCACTTCAATAAAAATCACTATTTCAA
NC-PP1_F	aggaagagagTTTTGTTGGTTTGTTTTAGGGTAGA
NC-PP1_R	cagtaatacgactcactatagggagaaggctAATCAAACTCCCTCTTCCATATACTC
NC-PP4_F	aggaagagagATATGGAAGAGGGAGTTTGATTTTT
NC-PP4_R	cagtaatacgactcactatagggagaaggctACCCCCTATACTCTTATTCTTTCAA

NP - NPSR1 promoter, PP - Primer Pair, NC - NPSR1 CTCF peak, F - Forward primer, R - Reverse primer
